# On Side-Chain Conformational Entropy of Proteins

**DOI:** 10.1371/journal.pcbi.0020168

**Published:** 2006-12-08

**Authors:** Jinfeng Zhang, Jun S Liu

**Affiliations:** Department of Statistics, Harvard University, Cambridge, Massachusetts, United States of America; University of California San Francisco, United States of America

## Abstract

The role of side-chain entropy (SCE) in protein folding has long been speculated about but is still not fully understood. Utilizing a newly developed Monte Carlo method, we conducted a systematic investigation of how the SCE relates to the size of the protein and how it differs among a protein's X-ray, NMR, and decoy structures. We estimated the SCE for a set of 675 nonhomologous proteins, and observed that there is a significant SCE for both exposed and buried residues for all these proteins—the contribution of buried residues approaches ∼40% of the overall SCE. Furthermore, the SCE can be quite different for structures with similar compactness or even similar conformations. As a striking example, we found that proteins' X-ray structures appear to pack more “cleverly” than their NMR or decoy counterparts in the sense of retaining higher SCE while achieving comparable compactness, which suggests that the SCE plays an important role in favouring native protein structures. By including a SCE term in a simple free energy function, we can significantly improve the discrimination of native protein structures from decoys.

## Synopsis

Side-chains of amino acids determine a protein's three-dimensional structure. The flexible nature of side-chains introduces a significant amount of conformational entropy associated with both protein folding and interactions. Despite many studies, the role that this side-chain entropy (SCE) plays in the process of folding and interactions has not been fully understood. Some basic questions about SCE have not been systematically studied. In this study, Zhang and Liu developed an efficient sequential Monte Carlo strategy to accurately estimate the SCE of proteins of arbitrary lengths with a given potential energy function. Using this novel tool, they studied how the SCE scales with the length of the protein, and how the SCE differs among a protein's X-ray, NMR, and decoy structures. They observed that X-ray structures pack more “smartly” than the corresponding decoy and NMR structures: with the same compactness, X-ray structures tend to have larger SCE. A combination of an SCE term with a contact potential energy significantly improved the discrimination between native and decoy structures. The implication of this study is that the SCE contributes so significantly to protein stability that it should be included explicitly in tasks such as structure prediction, protein design, and NMR structure refinement.

## Introduction

Side-chains of amino-acid residues encode the information governing a protein's three-dimensional fold. In a typical X-ray crystal structure, each residue's side-chain is represented by a fixed configuration, and most side-chain modelling methods assume that each buried side-chain takes only one fixed conformation among all possible rotameric states (rotamers) [1–4]. Recent studies, however, have shown that many different self-avoiding side-chain packing (called the side-chain conformation of a backbone structure henceforth) may exist for a given native backbone structure [5–7]. It is also well-recognized that the so-called “native protein structure” is an ensemble of structures instead of a single structure as normally seen from X-ray crystallography [8–11]. Ensemble properties of a protein are thus important for characterizing its structure and function.

Estimating ensemble properties such as entropy or free energy has been a long-standing difficult task in structure modelling and simulations [12,13]. In general, side-chain entropy (SCE) can be divided into the vibrational and the conformational [12]. Studies have shown that vibrational entropy is invariant in folded and unfolded states [14]. Therefore, most studies including ours focus on the estimation of conformational SCE [12]. Throughout this article, the term “SCE” actually refers to the conformational. Because of computational limitations, most of our current understanding of SCE is based on an aggregation of entropic effects such as rotamer counts of individual amino-acid residues [4,15–17], which has been shown to significantly overestimate the true SCE [18,19]. With the aid of a new Monte Carlo method, we can now accurately estimate the SCE of proteins based on a realistic model with all heavy atoms explicitly represented.

## Results

### A Large-Scale Analysis of Side-Chain Conformational Entropy

**Figure 1:**
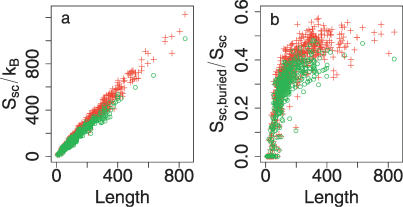
Side-Chain Entropy (SCE), *S_sc_*, of 675 Nonhomologous Proteins in the PDB (A) Side-chain entropy versus chain length. Two results with *α* = 0.6 (red crosses) and 0.8 (green circles) are shown. (B) Percentage of SCE contributed by buried residues versus chain length.

We computed SCE for a set of 675 nonhomologous proteins obtained from the PISCES database [20]. These proteins are selected under requirements that they have no missing residues; their structural resolutions are better than 1.6 Å; and no pairs have more than 20% sequence identity. The largest protein in the set has 839 residues. Figure 1A plots the SCE of proteins versus their chain lengths, showing that the SCE increases nearly linearly with the chain length. It also demonstrates that the SCE computation is insensitive to the use of two different scales for atom radius (see Methods). Furthermore, we estimated each individual residue's marginal SCE based on our weighted Monte Carlo samples, and observed that the fraction of SCE contributed by all the buried residues (defined as the one with less than 25% of its surface area accessible to solvent) of a protein approaches 40%–50% as the chain's length grows (Figure 1B).

### Side-Chain Entropy of the Native and Decoy Structures of Proteins

We considered all the 24 distinct monomeric proteins in five decoy sets (e.g., 4state_reduced, fisa, fisa_casp3, lattice_ssfit, and lmds) of the Decoys ‘R' Us database [21], in which each protein has a few hundred to ∼2,000 decoy structures and its native structure has been solved by X-ray crystallography. All decoy structures have been minimized using some physical force fields to reach a local energy minimum. Most of the decoy structures have large RMSD to the corresponding native structure (>3 Å).

**Figure 2:**
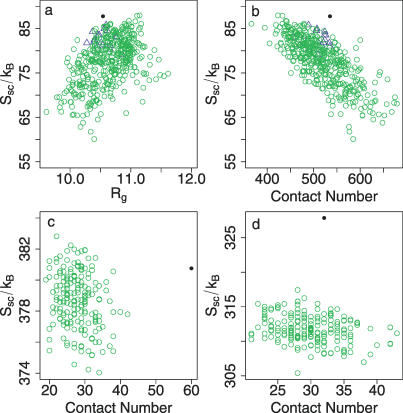
SCE of Native and Decoy Structures (A) SCE (*S_sc_*) versus the radius of gyration (*R_g_*). (B) SCE (*S_sc_*) versus the number of residue contacts (*N_c_*), for protein **1ctf** and its decoys from the 4state_reduced decoy set. (C) SCE (*S_sc_*) versus the number of interfacial contacts for protein–protein complex **1spb** and its decoys. (D) SCE (*S_sc_*) versus the number of interfacial contacts for protein–protein complex **1brc** and its decoys. The black dot is the native structure, blue triangles (<2.0 Å RMSD to the native structure) and green circles (>2.0 Å) are decoy structures. The SCE of protein complexes are calculated using *α* = 0.7 (see Methods).

We plot SCE (*S_sc_*) of the native and decoy structures of protein **1ctf** against the corresponding radii of gyration (*R_g_*) in Figure 2A, and against the number of residue contacts (*N_c_*) in Figure 2B. The measure *N_c_* has been suggested as a better compactness descriptor than the measure *R_g_* [22]. However, *R_g_* has been more commonly used than *N_c_* in the literature and, thus, makes it easier for us to compare with previous studies.

The result in Figure 2 is surprising. First, for structures with similar compactness measured by *R_g_,* their *S_sc_* can differ by more than 20 in *k_B_* units, which corresponds to 11.9 kcal/mol of free energy at 300 K. Considering that the average stability of proteins is at −5 to −20 kcal/mol, this difference is huge. Second, the native structure has a higher *S_sc_* than all decoy structures with similar compactness. A line can be drawn on the *R_g_*−*S_sc_* plane to perfectly separate the native and decoy structures. Among the 24 proteins we studied (Protocol S1), half of these proteins show similar distributions to that of **1ctf**. The other dozen proteins possess either disulfide bonds, metal binding sites, or interacting sites with other molecules, which impose additional constraints on their native structures that lead to lowered SCE [23]. In contrast, most decoy structures do not satisfy these constraints.

We observed a similar phenomenon for dimeric protein complexes in the decoy set generated by the Rosetta program [24]. Two representative examples are shown in Figure 2C and 2D, in which the native protein complex **1spb** has more interfacial contacts than all the decoys but with comparable SCE, and **1brc** has much higher SCE than all the decoys, but with a comparable number of interfacial contacts.

### Side-Chain Entropy of X-Ray and NMR Structures

**Figure 3:**
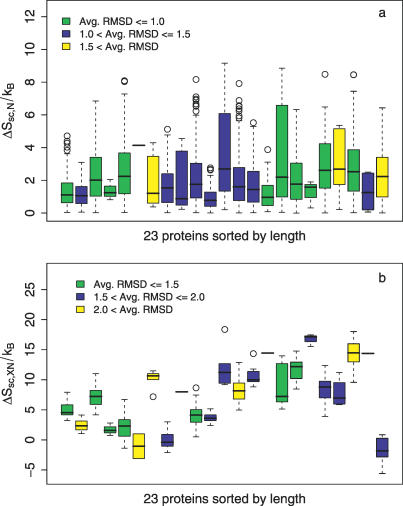
SCE of NMR and X-Ray Structures (A) Box plot for distributions of the absolute pairwise SCE difference (|Δ*S_N_*|) of NMR structures of 23 proteins. Different coloured boxes indicate different ranges of average RMSDs of the structure pairs. (B) Box plot for distributions of the SCE difference between X-ray and NMR structures (Δ*S_XN_*) for 23 proteins. Different colours indicate different ranges of average RMSDs of the X-ray and NMR structure pairs. For proteins 1btv, 1vre, and 1ah2, *α* = 0.7 was used for both X-ray and NMR structures.

We chose 23 out of the 60 proteins in [25] (names are given in the legend of Figure 3) under requirements that multiple NMR structures are available for each protein, and that NMR and X-ray structures correspond to the same sequence. The distribution of |Δ*S_N_ | = |S_sc,NMR2_* − *S_sc,NMR1_|*, the absolute SCE difference between all pairs of NMR structures for each of these proteins, is shown in Figure 3A. Although the majority of these differences is small, there are a significant number of pairs with |Δ*S_N_*| more than 5 *k_B_* units, corresponding to 3 kcal/mol of free energy at 300 K.

**Figure 4:**
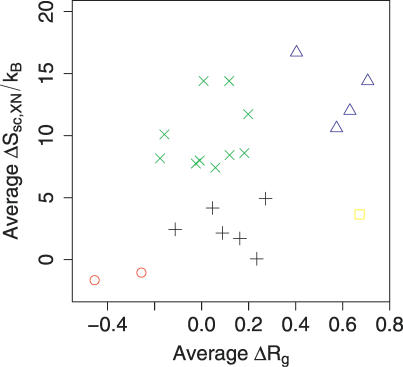
SCE of NMR and X-Ray Structures versus Rg Average SCE difference between X-ray and NMR structures (Δ*S_XN_*) versus the average difference of radius of gyration between X-ray and NMR backbones (Δ*R_g_*) for the 23 proteins. ×, proteins whose X-ray structures have much higher SCE than but similar *R_g_* to the corresponding NMR structures. Δ, proteins whose X-ray structures gain considerable SCE by packing a little looser. ○, proteins whose X-ray structures pack tighter than NMR structures but with comparable SCE. +, small proteins of which both Δ*R_g_* and Δ*S_XN_* are small.

The SCE difference between X-ray and NMR structures, Δ*S_XN_* = *S_sc,X-ray_* − *S_sc,NMR_,* displays a much different behaviour. As shown in Figure 3B, magnitudes of Δ*S_XN_* between proteins' X-ray structure and their corresponding multiple NMR structures are much larger than |Δ*S_N_* |'s (2 versus 8 *k_B_* unit on average). Although each chosen X-ray structure is very similar to its corresponding NMR structures with small RMSD [25], X-ray structures generally have higher SCE than the corresponding NMR structures. To see how this is related to their packing, we show in Figure 4 the average Δ*S_XN_* of a protein versus Δ*R_g_* = *R_g_*,*_X-ray_* − *R_g,NMR_,* the average difference of the radius of gyration of backbone atoms between X-ray and NMR structures, for all the 23 proteins. Clearly, X-ray structures have comparable *R_g_* to the corresponding NMR structures. For many proteins (“×” in Figure 4), their X-ray structures have much higher SCE than the corresponding NMR structures with similar *R_g_*. Some proteins' X-ray structures (“Δ”) gain considerable SCE by packing a little looser. Two X-ray structures (“○”) pack tighter than NMR structures but with comparable SCE. Small proteins (“+”) tend to have small Δ*R_g_* and Δ*S_XN_*, while large proteins tend to have large Δ*S_XN_* (see also Figure 3). This is expected since NMR experiments tend to be more accurate for small proteins.

### Incorporation of Side-Chain Entropy in Free Energy Functions

Since native structures tend to have higher SCE than computer-generated decoys at the same level of compactness, incorporating SCE into free energy functions should improve modelling accuracy. We tested this idea on all 24 distinct proteins and their decoys in the Decoys ‘R' Us database. We use a statistical contact potential [26] based on the pairwise distances of C_β_ atoms, which can be easily computed from a protein's backbone structure. Following the equation of Gibbs free energy, the free energy of ensemble structures represented by a backbone structure is defined as: *G_bb_* = *H_bb_* – *TS_SC_,* where *H_bb_* is the potential energy defined by the backbone conformation, *S_sc_* is the side-chain entropy, and *T* is the temperature. Since we use here a statistical potential, temperature *T* has no physical meaning and can be freely adjusted. We set *T* to 1 in this study without optimization. We use the rank of the native structure among all the decoys to evaluate the discrimination performance. Table 1 shows that for most proteins, the measures based on free energy *G_bb_* significantly improved the discrimination power compared with those using potential energy *H_bb_*. For a few proteins, the discrimination performances under *G_bb_* and *H_bb_* are comparable, and in only one case *G_bb_* performed slightly worse than *H_bb_*. It is possible that some proteins are stabilized mainly by enthalpy and other entropic terms instead of by side-chain conformational entropy. For example, the energy of a couple of disulfide bonds may be enough to stabilize a small protein so that other factors become insignificant.

**Table 1:**
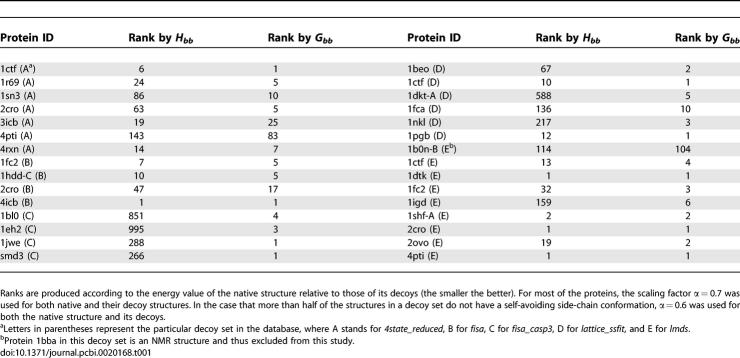
Discrimination of Native Structures Using a Free Energy Function

We note here that an all-atom potential function, which differentiates different side-chain conformations, can also be accommodated by our Monte Carlo method. In particular, free energy *G_bb_* can be estimated using the formula *G_bb_* = −*k_B_T*ln(*Q_bb_*), where *Q_bb_* is the partition function of the ensemble side-chain conformations of a backbone structure, which can be estimated by our Monte Carlo method.

## Discussion

In this study, we systematically investigated the SCE of a large set of protein structures and its difference among X-ray, NMR, and decoy structures. Our findings do not contradict the traditional view that SCE is an opposing factor for protein folding from extended states to compact native states, but our findings on the systematic difference of the SCE among the folded conformations with similar compactness suggest that the SCE plays an important role in protein stability and should be included in tasks such as protein structure prediction, protein design, and NMR structure refinement.

The Nuts-and-Bolts model states that proteins pack quite randomly, thus giving rise to many internal voids [19,22,27,28]. In contrast, the Jigsaw-Puzzle model alleges that proteins pack like a jigsaw puzzle with side-chains closely interlocked [29–33]. It is conceivable that side-chain packing in protein cores is not completely random, as some regularities and specific residue interactions have been observed [32,33]. However, such specific interactions are sparse among all interacting residue pairs [34]. Our observation that buried residues of a protein contribute significantly to its overall SCE suggests that the interior of a protein's native structure is unlikely to pack in a jigsaw-puzzle mode. However, we also found that the SCE of individual buried residues vary greatly, with some having comparable entropy to exposed ones while others have almost zero entropy, which is consistent with observed local packing in proteins [35]. This indicates that the packing of the protein core is likely heterogeneous, with parts forming a jigsaw puzzle to gain specificity and other parts resembling nuts and bolts to maintain entropy and gain robustness against mutations.

Structures solved by X-ray crystallography are generally more reliable than the corresponding NMR structures, which lack the quality measurement for solved structures. It has been found that NMR structures tend to pack poorly [36]. Such poor packing is mainly due to the nature of experimental data and computational methods employed instead of a reflection of the difference between the solution and crystal states. Indeed, experimental NMR observables agree better with structures calculated from high-resolution crystals than those from the corresponding NMR structures [37]. Our findings suggest that the SCE difference found between X-ray and NMR structures may account for some of the poor packing of NMR structures, and thus, incorporating SCE in the energy functions used in computational methods of NMR experiments, may improve the quality of NMR structures.

Both decoy and NMR structures were obtained by structural optimization under some potential functions. The backbone conformational entropy has been suggested as a stabilizing factor for native proteins. [38] Observations made in this study indicate that ignoring SCE by those optimization techniques produces significant deviations from characteristic packing and interaction of native proteins, which suggest that atom-level modelling of protein structures and interactions should take approaches with more emphasis on ensemble sampling rather than on optimization. Our preliminary study on the incorporation of SCE in an empirical free energy function shows a significant improvement in discrimination of native structure against decoys.

We used in SCE estimation a very simplified energy function, which focuses only on the excluded volume effect. It is somewhat surprising to us that, just with excluded volume effect, the SCE can already differentiate well native X-ray structures from NMR and decoy ones. We also experimented with another energy function considering rotamer probabilities, which reduces the SCE by 10% on average, and observed that the results reported here hold well. It remains to be seen how the reported results will be affected when a more realistic potential energy function is used. For example, if a Van der Waals interaction term is to be added, the discrete rotamer formulation adopted in this article's research has to be adequately refined so as to accommodate the continuous nature of the protein side-chain positions. Otherwise, the SCE could be seriously distorted when a few atoms are not placed very well due to the discrete nature of side-chain rotamers.

Interfacial regions in protein–protein complexes have been shown to be less flexible than other parts of the protein surface [23]. It has also been suggested that conserved polar residues at the binding interfaces have higher rigidity so that the entropic cost is minimized on binding, whereas surrounding residues form a flexible cushion [39]. These studies suggest that conformational entropy may play important roles in protein interactions. A recent study has assessed prediction difficulties of protein–protein complexes based on CAPRI [40] results, which indicated that one type of difficult complex has a small interface area and a weak binding energy [41]. Existing computational docking algorithms typically favor interaction conformations with large interface areas, thus producing many false positives for this type of complex. As shown in Figure 2, we believe that an energy function incorporating an SCE term should improve the prediction accuracy of this and any other type of protein complex in which SCE contributes significantly in the binding free energy.

## Materials and Methods

### Side-chain modelling.

Each residue's side-chain conformation is modelled as a rotamer with a finite number of discrete states [42]. The rotamer library used is developed by Lovell et al. [43], as recommended by Dunbrack [42] for the study of entropy. The rotamer library of Dunbrack and Cohen [44] was also applied to some of the proteins studied here and similar results were observed. To account for the excluded volume effect (or self-avoiding requirement), we took the approach of Kussell et al. [6], in which a pair of atoms *i* and *j* is considered to be a hard clash if *r_ij_* < *α* × (*r*_0_(*i*) + *r*_0_(*j*)), where *r_ij_* is their distance, *α* is a scaling coefficient to account for the discrete nature of side-chain rotamers, and *r*_0_(*i*) and *r*_0_(*j*) are the van der Waals radii of the two atoms. We tested three *α* values at 0.6, 0.7, and 0.8, respectively, and found that they gave qualitatively similar results (Figure 2). Lower *α* values give higher entropy and diminish the side-chain entropy differences among different structures, whereas higher *α* values give lower entropy and cause some structures to have no valid self-avoiding side-chain conformations, which were discarded in the analysis. All results on the comparison of X-ray, decoy, and NMR structures were obtained with *α* equal to 0.8, unless otherwise stated.

The SCE is defined as: 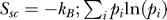 , where *k_B_* is the Boltzmann constant and 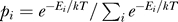 is the probability of a self-avoiding side-chain conformation. When the *p_i_*'s are all equal or *T* is very high, we have *S* = *k_B_*ln(*n_sc_*), where *n_sc_* is the number of self-avoiding side-chain conformations for the given backbone structure. The compactness measurement *N_c_* is defined as number of pairwise C_β_ (or C_a_ of Glycine) atoms with their distance of less than 7.5 Å.

### Sequential Monte Carlo method.

The Sequential Monte Carlo method (SMC) is a generalization of the Rosenbluths' chain growth method [45] and has been applied previously in studying problems ranging from protein-packing behaviour, effect of amino acid chirality, side-chain flexibility, protein folding, and near-native structures of proteins [19,22,46–48]. In this work, we made two design modifications to further improve the SMC's efficiency: (a) we make use of a recently developed stratified resampling technique [19,47], and (b) we take advantage of the fact that the sampling order of each residue's conformation can be arranged arbitrarily. A brief description on the method is given below. More details about the general method can be found in [19,22,46,48].

Given a fixed backbone structure with *n* residues, a realization of side-chain placement can be represented as *S_n_* = (*r_1_*,…*r_n_*), where *n* is the length of the protein sequence, *r_i_*∈ 1… *M_i_* is the rotameric state of residue *i* with *M_i_* being the number of rotamers at residue *i*. Let Ω*_n_* be the space of all self-avoiding side-chain conformations with the given backbone structure. We are interested in estimating:

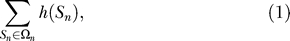
where *h*(*S_n_*) is a given function. This can be achieved by the importance sampling formula,

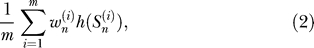
where each 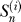 is sampled with probability 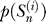 and 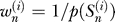 is its weight.

Conformation 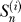 and its associated weight 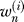 are constructed by stochastically placing the side-chain rotamer of every residue sequentially. Once the side-chain of a residue is sampled, it is regarded as fixed and thus reduces the degrees of freedom for side-chain placements of future residues. Initially (step 0), we set the weight 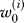 to 1 and place no side-chains on the backbone. At step *t +* 1, we check the environment of every residue of the chain whose side-chain has not been placed. Then, we place the side-chain for the residue with the most restrictive environment by sampling a rotamer valid for this residue from a given distribution. The weight of the chain is updated to 
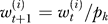
 , where *p_k_* is the probability of sampling rotamer *k* for this residue. This probability is calculated as 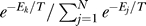 , where *E_k_* is the energy of rotamer *k* (see below for the details of the energy functions used) and *N* is the total number of valid side-chain rotamers at the residue being sampled. After the placement of this side-chain, environments of all other unsettled side-chains are updated. If no valid self-avoiding rotamer can be found for a residue, then the weight of the chain is set to zero and a stratified resampling procedure is performed to replace the dead chain by an existing chain with large weights [19,22,47].

Using the weights computed recursively as above, we can estimate the partition function 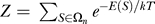 by Equation 2 with function *h*(*S*) = *e* ^−*E*(*S*)/*kT*^. The SCE, *S_SC_*, can also be estimated by Equation 2 using function *h*(*S*) = −*p*(S)ln(*p*(*S*)), where *p*(*S*) = *e* ^−*E*(*S*)/*kT*^ / *Z* is the Boltzmann probability of conformation *S*. Since we do not know the true partition function *Z,* we replace it by its importance sampling estimate. The estimated partition function can also be used to estimate free energy. In this study, two potential functions were used: *E* = *E_0_*, a constant, and 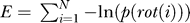 , where *N* is number of residues and *p*(*rot*(*i*)) is the database derived probability of the rotamer sampled at residue *i*. All figures shown in this paper are the results from using *E* = *E_0_*. We also studied SCE using the second potential function for some of the proteins and found that it gave qualitatively similar results to those from using *E* = *E_0_*.

The SCE of an individual residue *k* is: 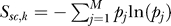 , where *p_j_* is the probability of rotamer *j* and *M* is the number of all possible rotamers at residue *k*. We estimate *p_j_* at residue *k* as 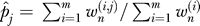 , where *w_n_^(i,j)^* is the weight of sample *i* with its residue *k* taking the rotamer state *j*.

### Performance of SMC in estimation of side-chain entropy.

**Figure 5:**
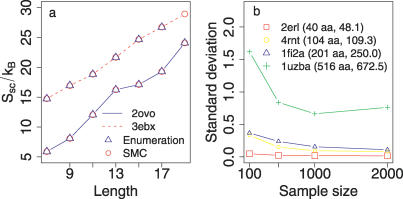
Performance of the Sequential Monte Carlo Method (A) Comparison of the SMC estimation with exhaustive enumeration for fragments of proteins 2ovo and 3ebx. (B) Standard deviation of the SMC estimation for four different sample sizes, 100, 500, 1,000, and 2,000, respectively, calculated from 20 independent SMC runs. The first number in each parentheses pair is the number of residues of the protein, and the second number the average SCE of 20 runs with 1,000 samples in each run.

We selected two proteins, 2ovo and 3ebx, and enumerated all the self-avoiding side-chain conformations, which give rise to exact SCEs for their backbone fragments of various lengths from residue 1 to 19. We then used SMC to estimate SCEs for these fragments and compared with the exact answers. As seen from Figure 5A, the estimates using SMC are indistinguishable from those obtained by exhaustive enumeration. For example, the total number of self-avoiding side-chain conformations for the fragment of 3ebx, residue 1–17, is 396,325,923,840, and our SMC estimate is 4.01 × 10^11^ with the Monte Carlo sample size *M* = 1,000. Figure 5B shows the standard deviations of these estimates against the sample size *M* used by SMC. We found that a single run of SMC with *M* = 1,000 is enough to give accurate estimates of the SCE for all the proteins we studied.

The running time of SMC with *M* = 1,000 samples and α = 0.6, on a Linux machine with a CPU of 1.4 GHz, was 3.1 s for protein 4rnt (104 residues); 6.4 s for protein 1svn (269 residues); and 81 seconds for protein 1epw (1,287 residues), the longest protein we have tried.

## Supporting Information

Protocol S1 Side-Chain Entropy and Packing of Native and Decoy Structures (3.3 MB PDF)

### Accession Numbers

PDB names of NMR structures of the 23 proteins in Figure 3A are (with PDB names of X-ray structures and protein lengths in parentheses): 1erc (2erl, 40), 1tur (2ovo, 56), 1f2g (1fxd, 58), 1fra (3ebx, 62), 1r63 (1r69, 63), 3mef (1mjc, 69), 2ait (1hoe, 74), 1cdn (3icb, 75), 2pac (451c, 82), 1hdn (1cm2, 85), 2abd (1hb6, 86), 1afh (1mzl, 93), 1bmw (1who, 94), 1ygw (4rnt, 104), 1it1 (2cdv, 107), 1xoa (2tir, 108), 2aas (1kf5, 124), 1pfl (1fil, 139), 1vre (1jf4, 147), 1rch (1rbv, 155), 1eq0 (1hka, 158), 1btv (1bv1, 159), 1ah2 (1svn, 269).

## Abbreviations

rotamers: rotameric states
SCE: side-chain entropy
SMC: Sequential Monte Carlo
