# Supplementary material for: On Side-Chain Conformational Entropy of Proteins
Source: PLoS Comput Biol. 2006 Dec 8;2(12):e168. doi: 10.1371/journal.pcbi.0020168 (PMC1676032; doi:10.1371/journal.pcbi.0020168)
Supplement: Supplementary file 1 [file pcbi.0020168.sd001.pdf]

Supplementary material for the paper
On side-chain conformational entropy of proteins
Jinfeng Zhang and Jun S. Liu

I. Side-chain entropy versus radius of gyration (R_g) and number of contacts (N_c) for X-ray protein structures and their decoys of monomeric proteins. Structures with no self-avoiding side-chain conformation are omitted. Natives are represented by red dots and decoys empty green circles. Y-axis is side-chain entropy and x-axis is R_g and N_c.

1. 4state_reduced decoy set

1r69

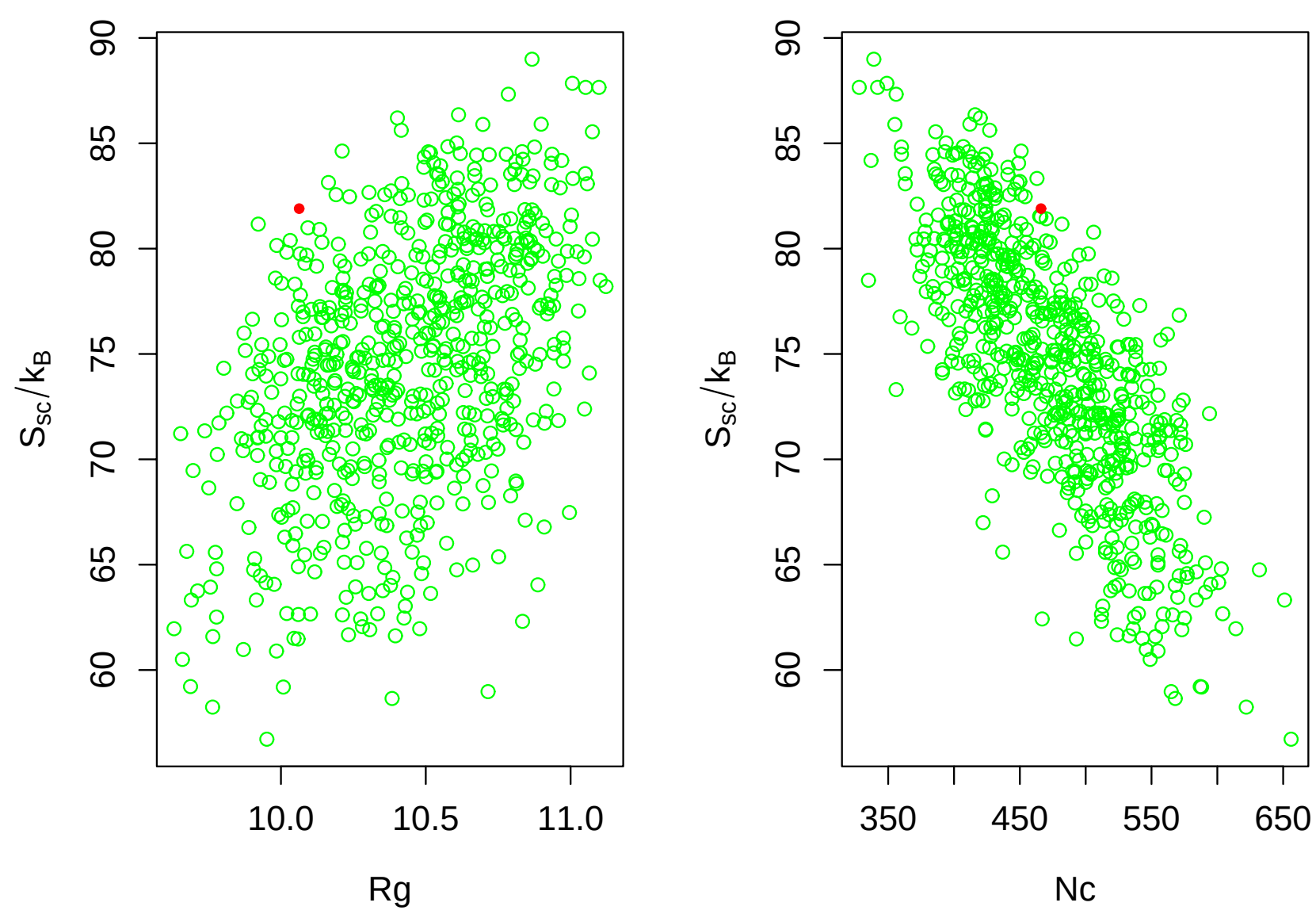

1sn3

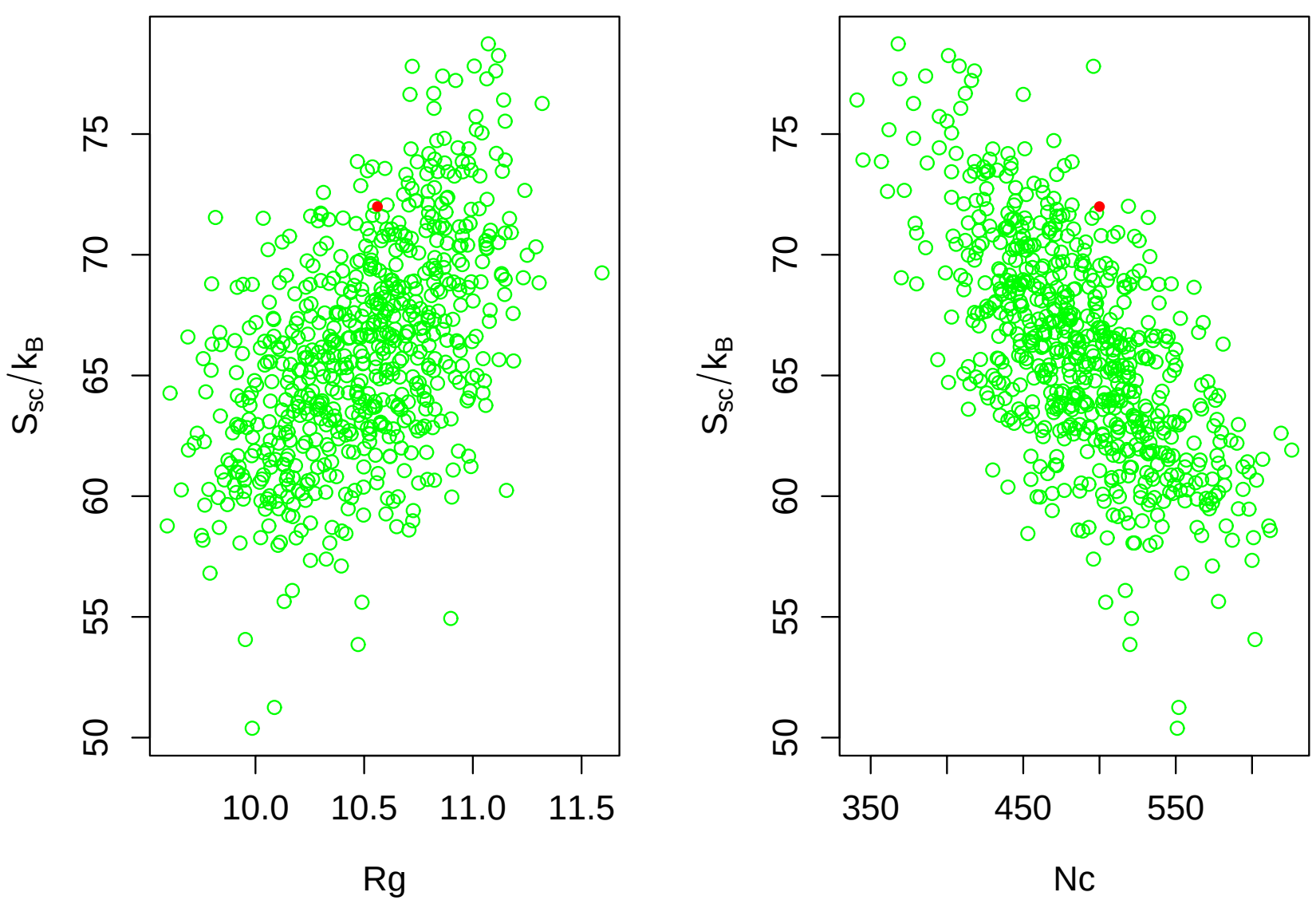

2cro

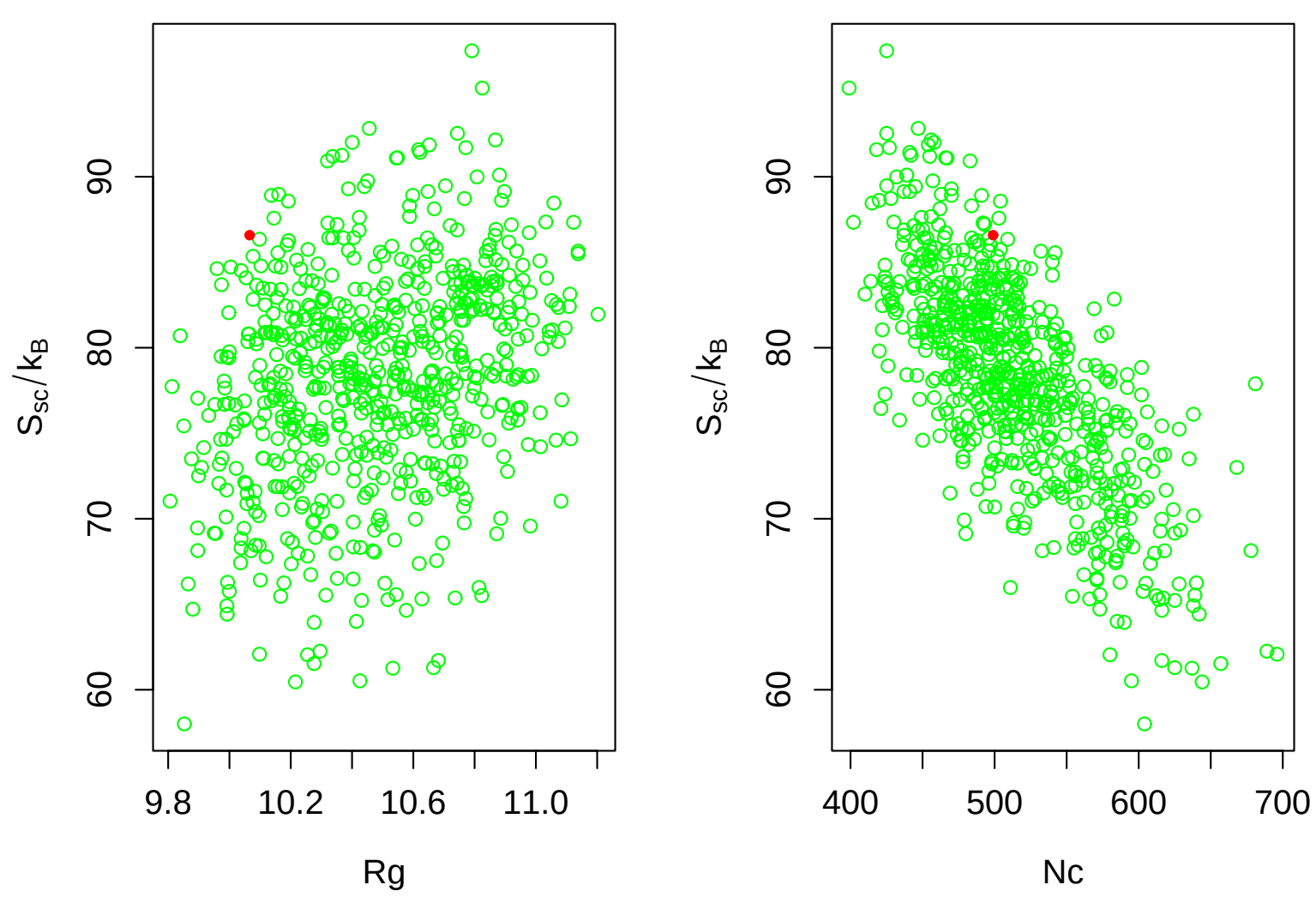

3icb (Metal binding protein)

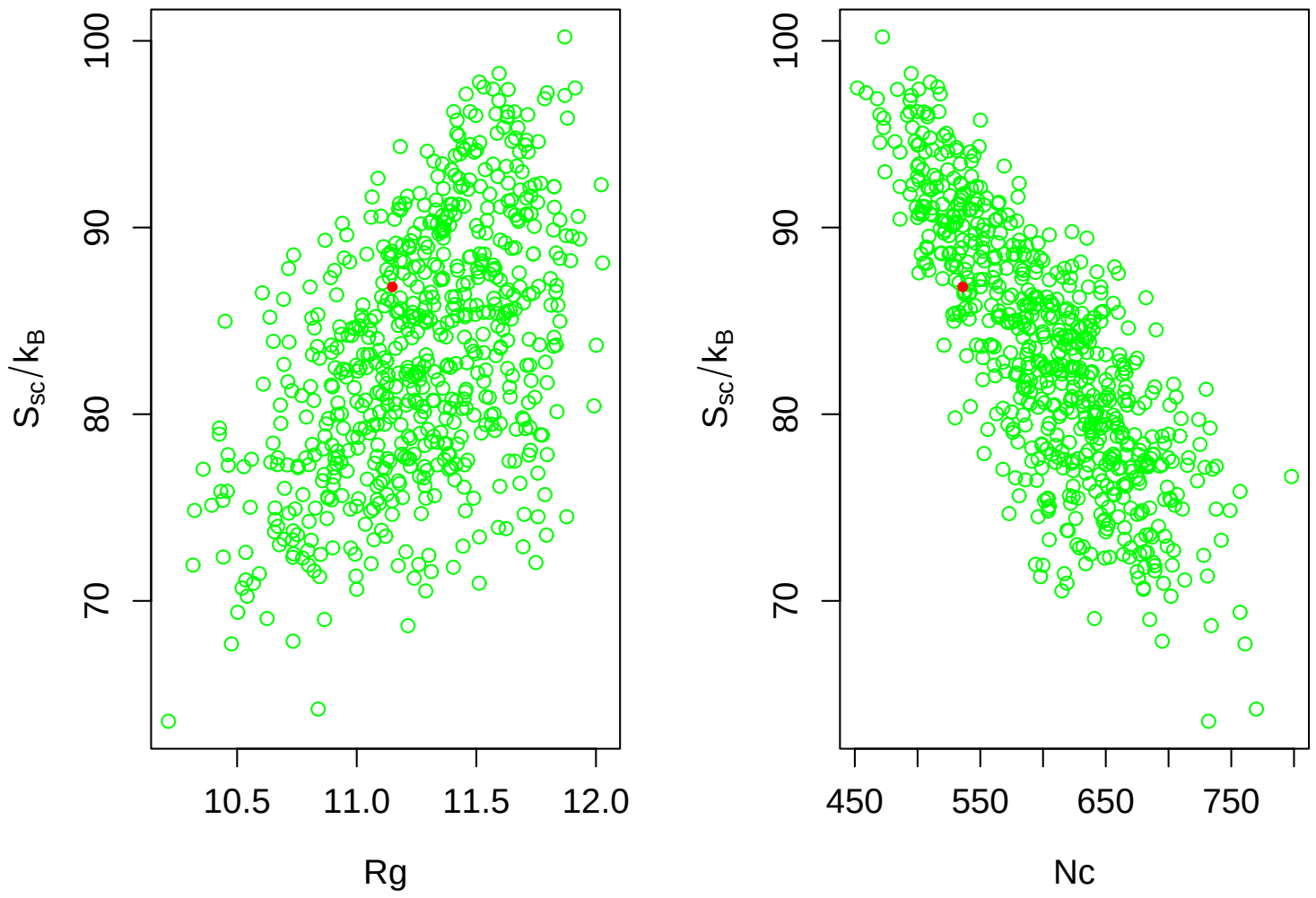

4pti (Protein with disulfide bonds)

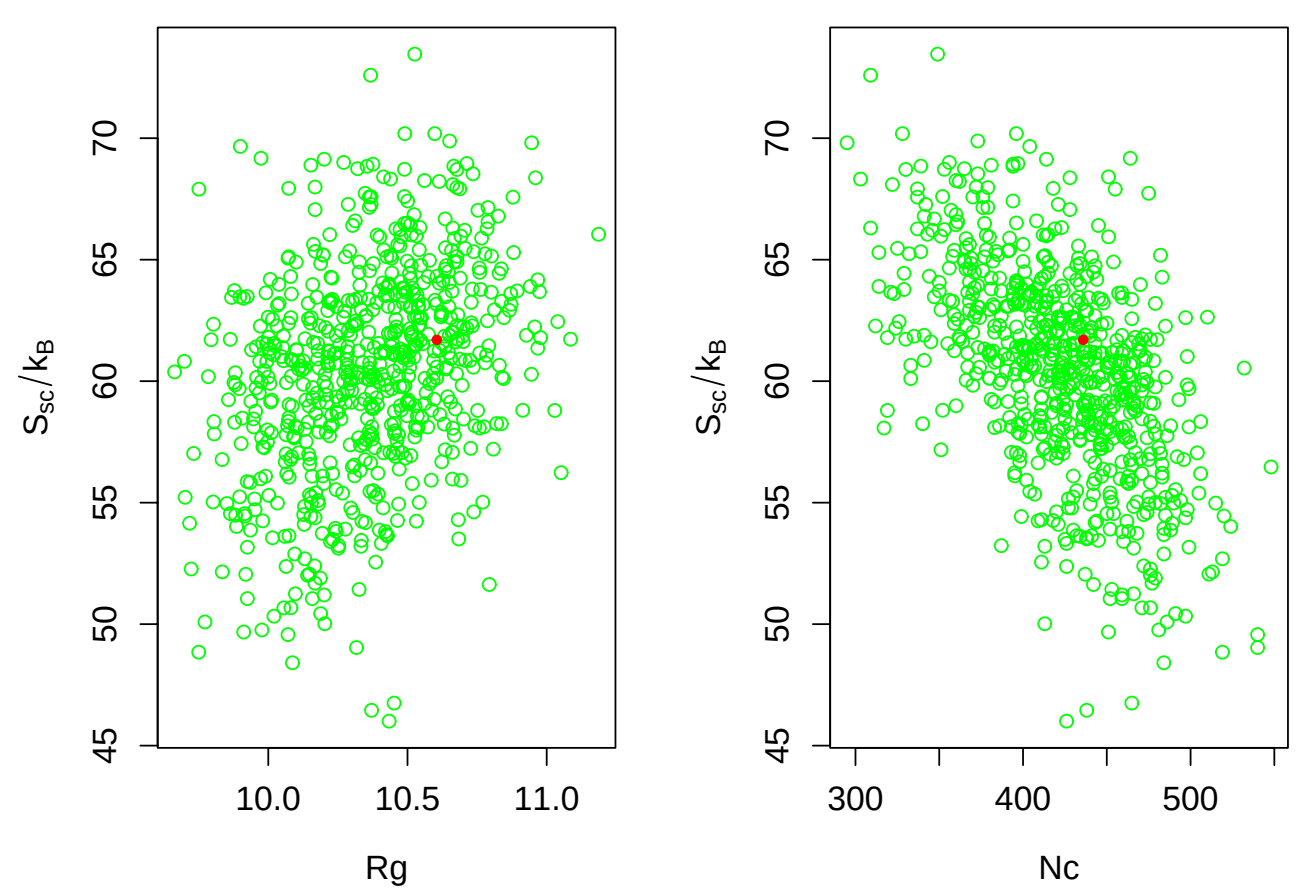

4rxn (Metal binding protein)

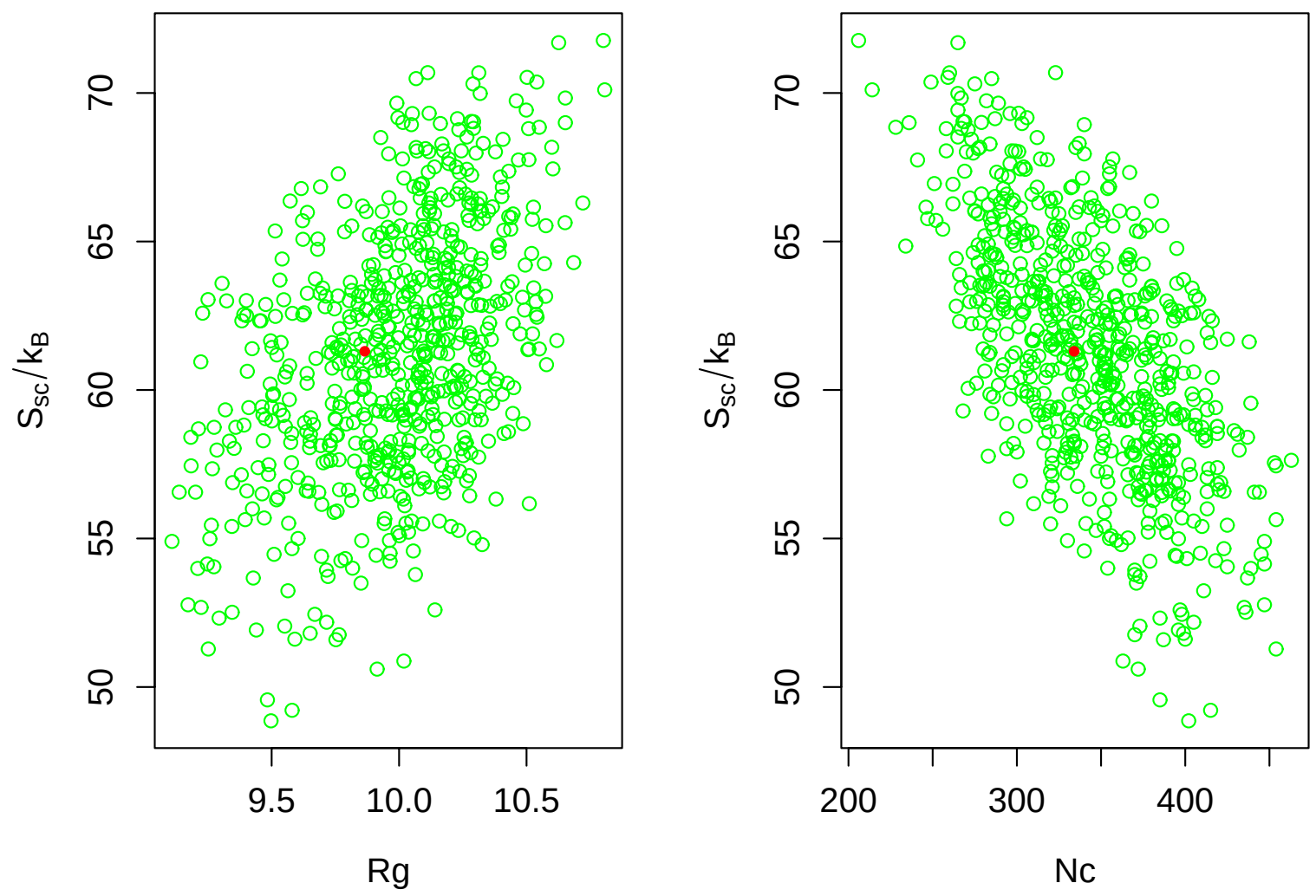

2. fisa

1fc2 (Protein interacting with another protein)

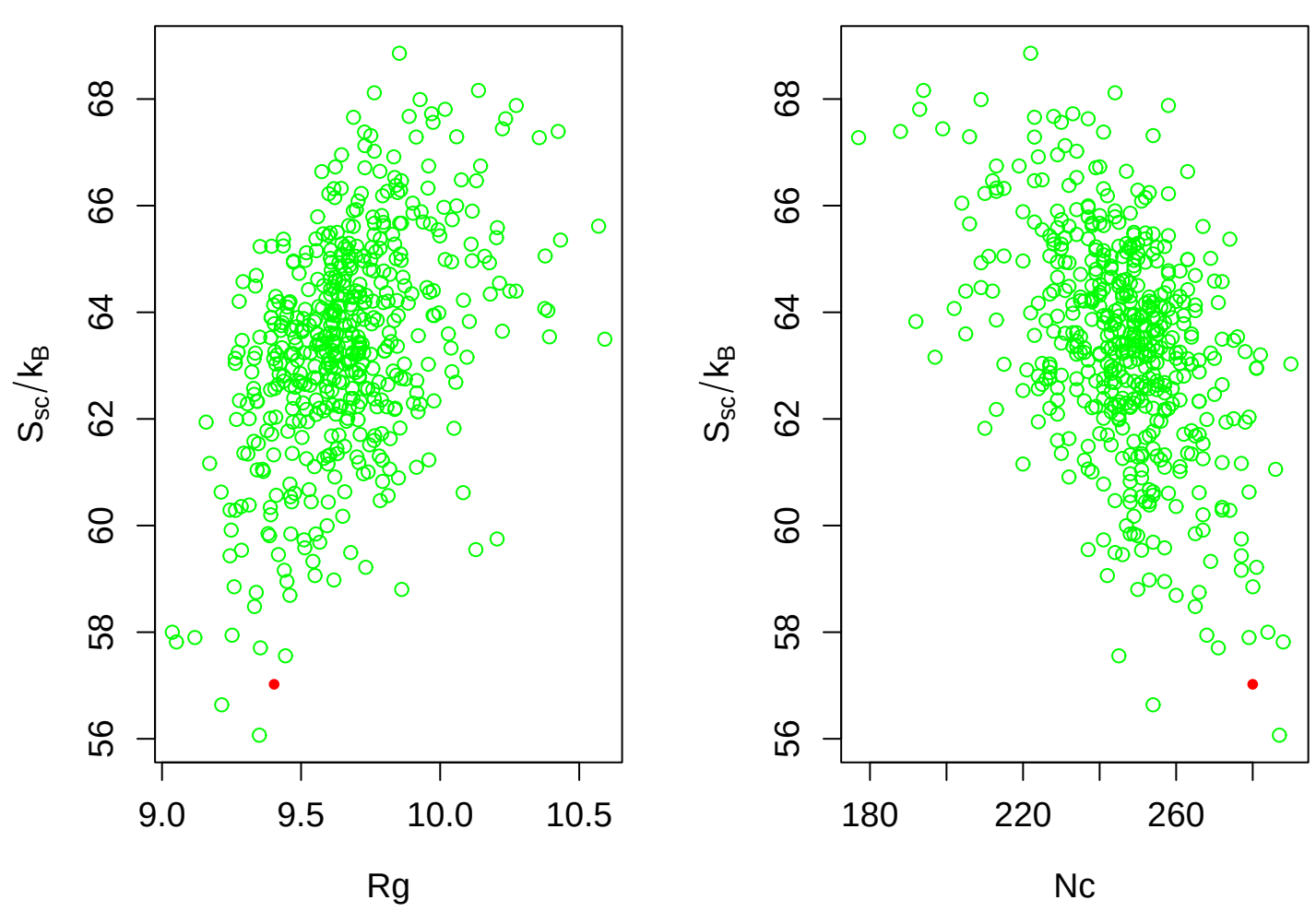

1hdd-C (Protein interacting with another protein)

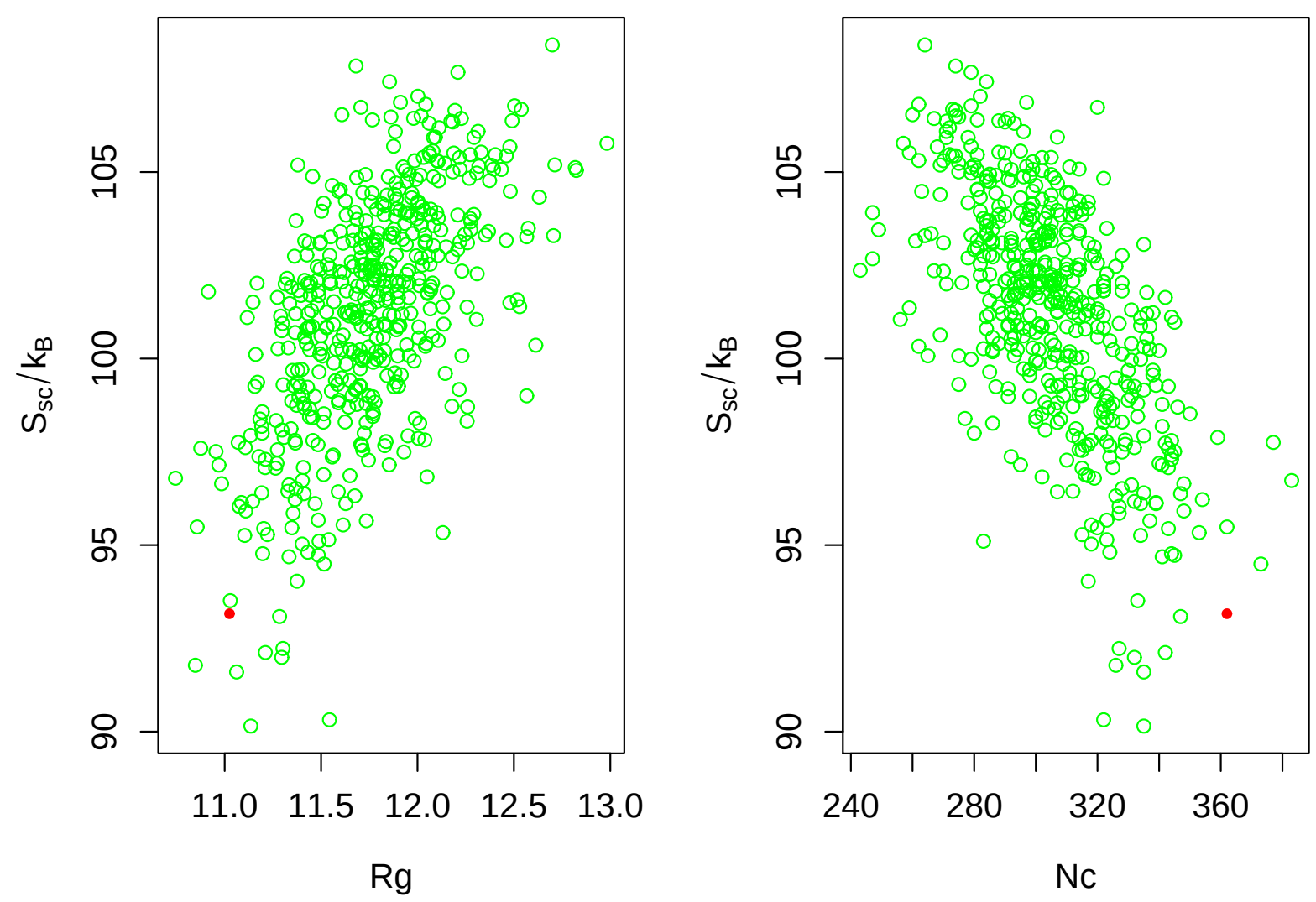

2cro

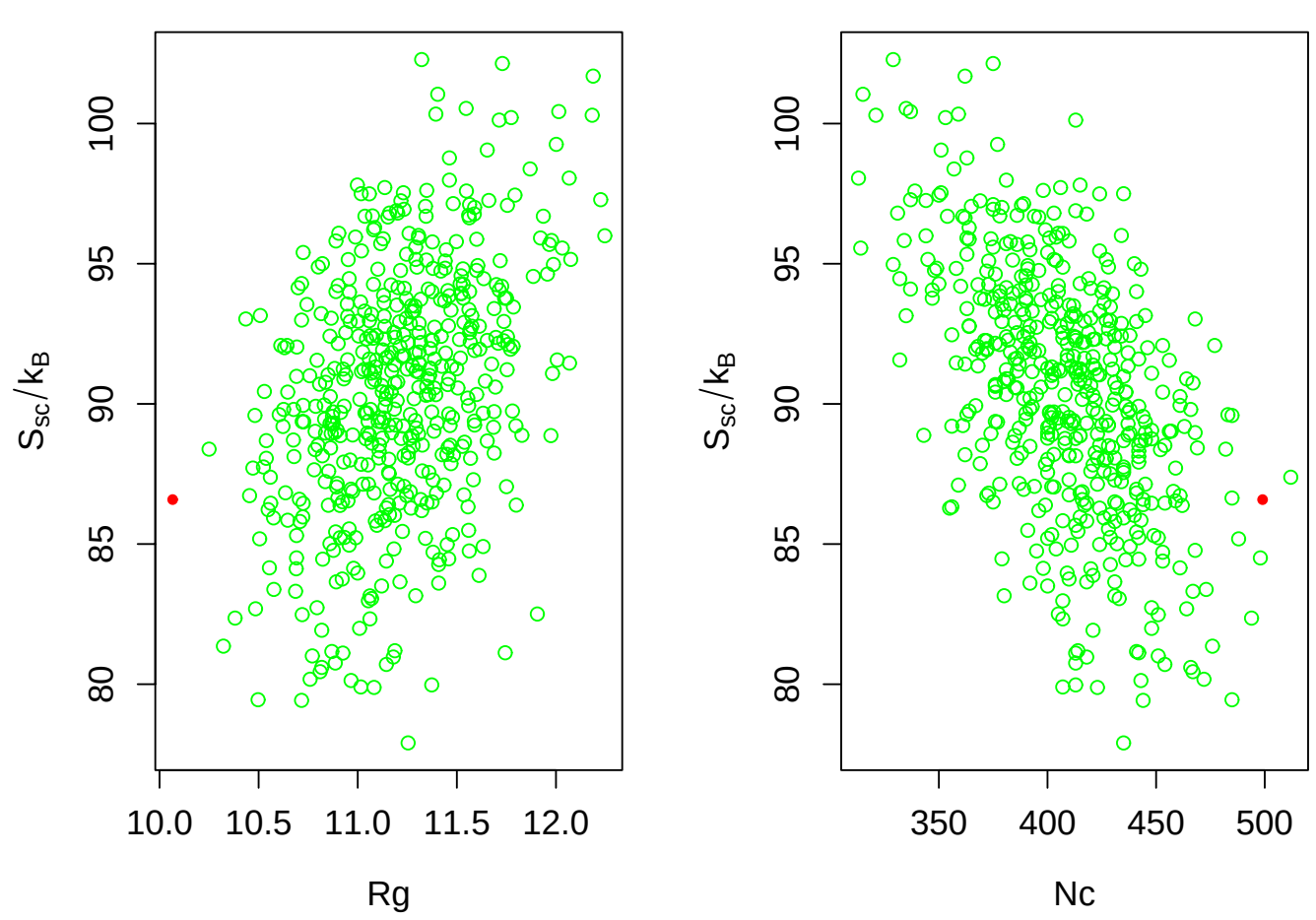

3. fisa_casp3

1bg8-A (Protein with Disulfide bonds)

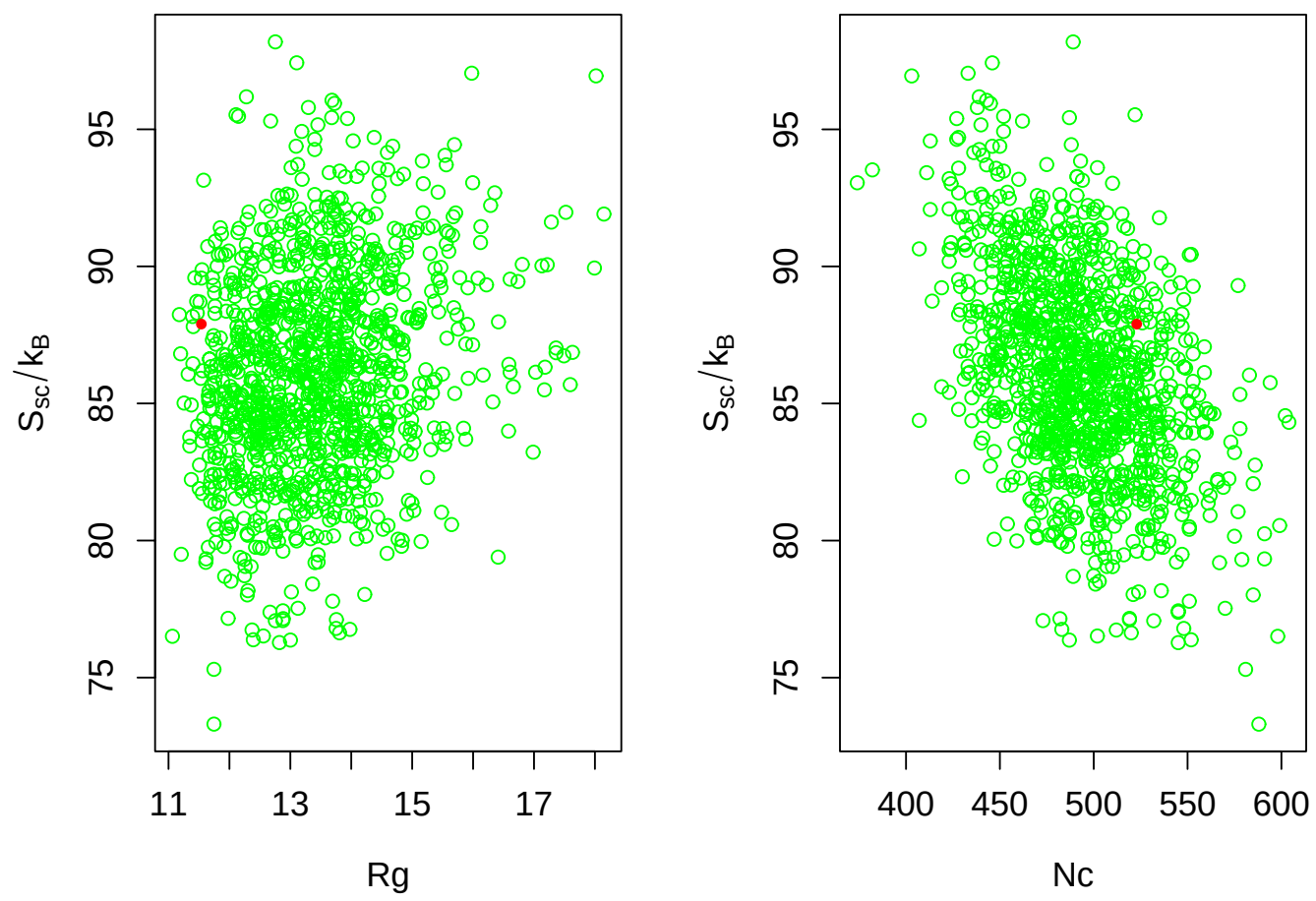

1bl0

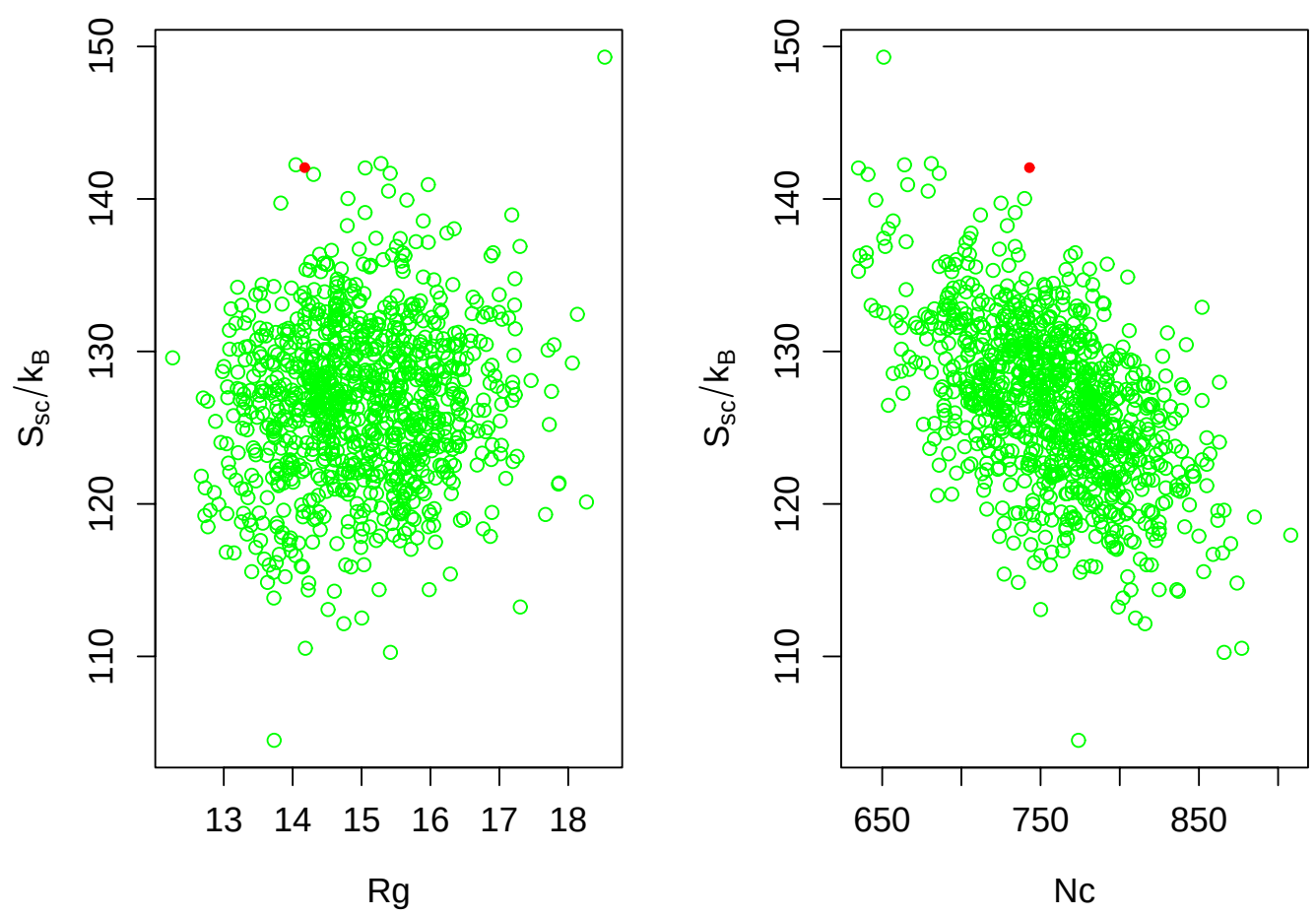

1eh2 (Metal binding protein)

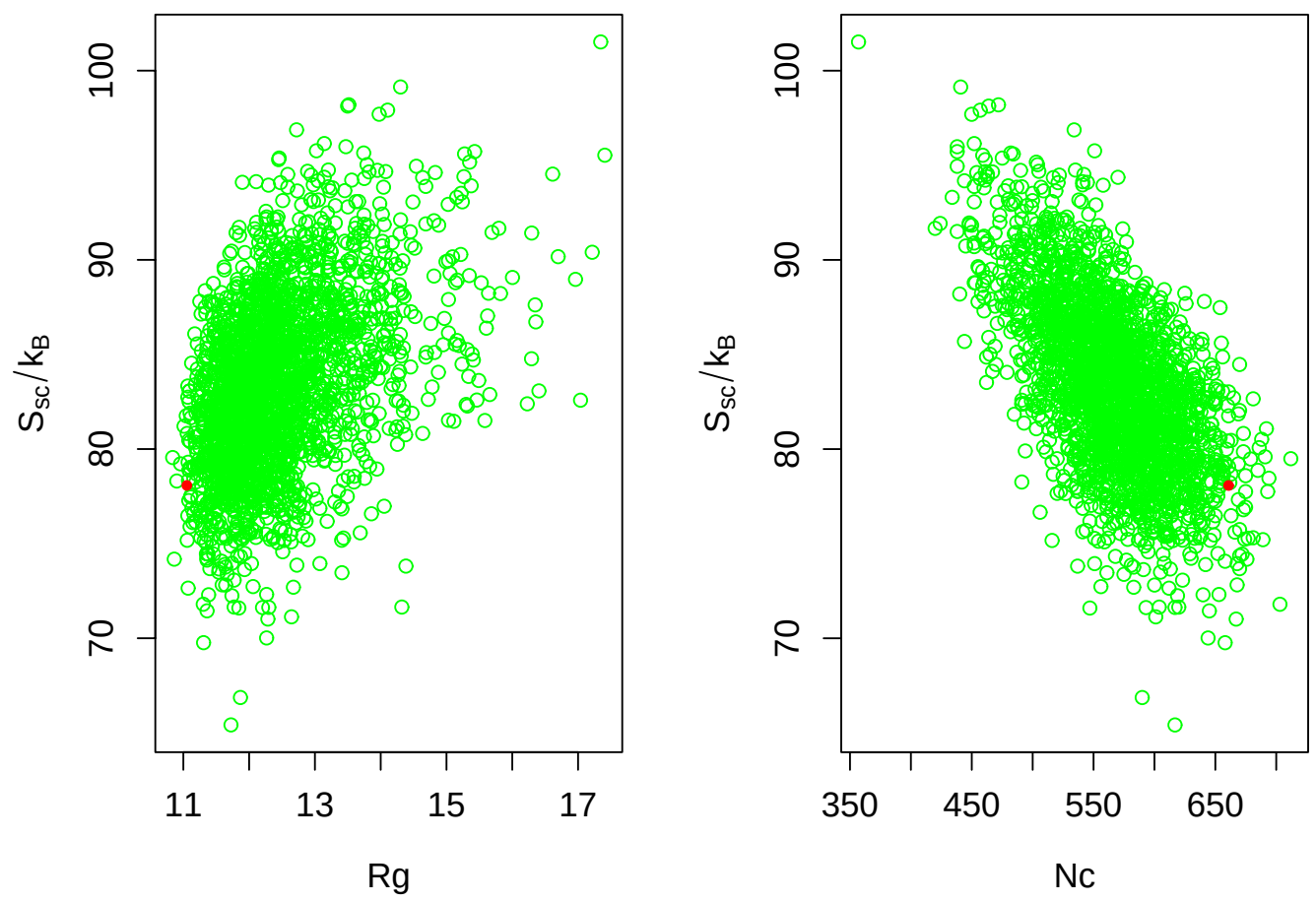

smd3

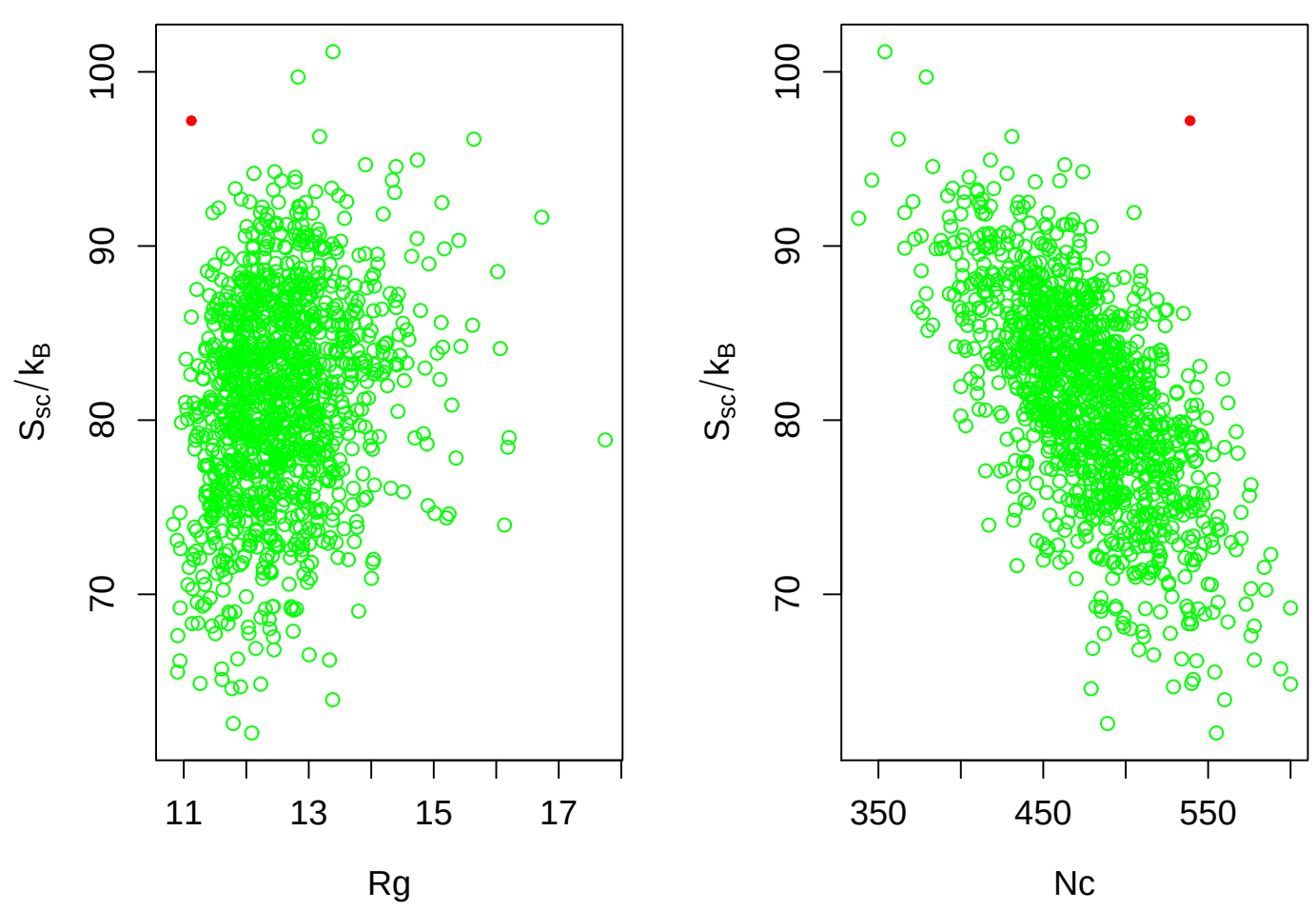

4. lattice_ssfit

1ctf

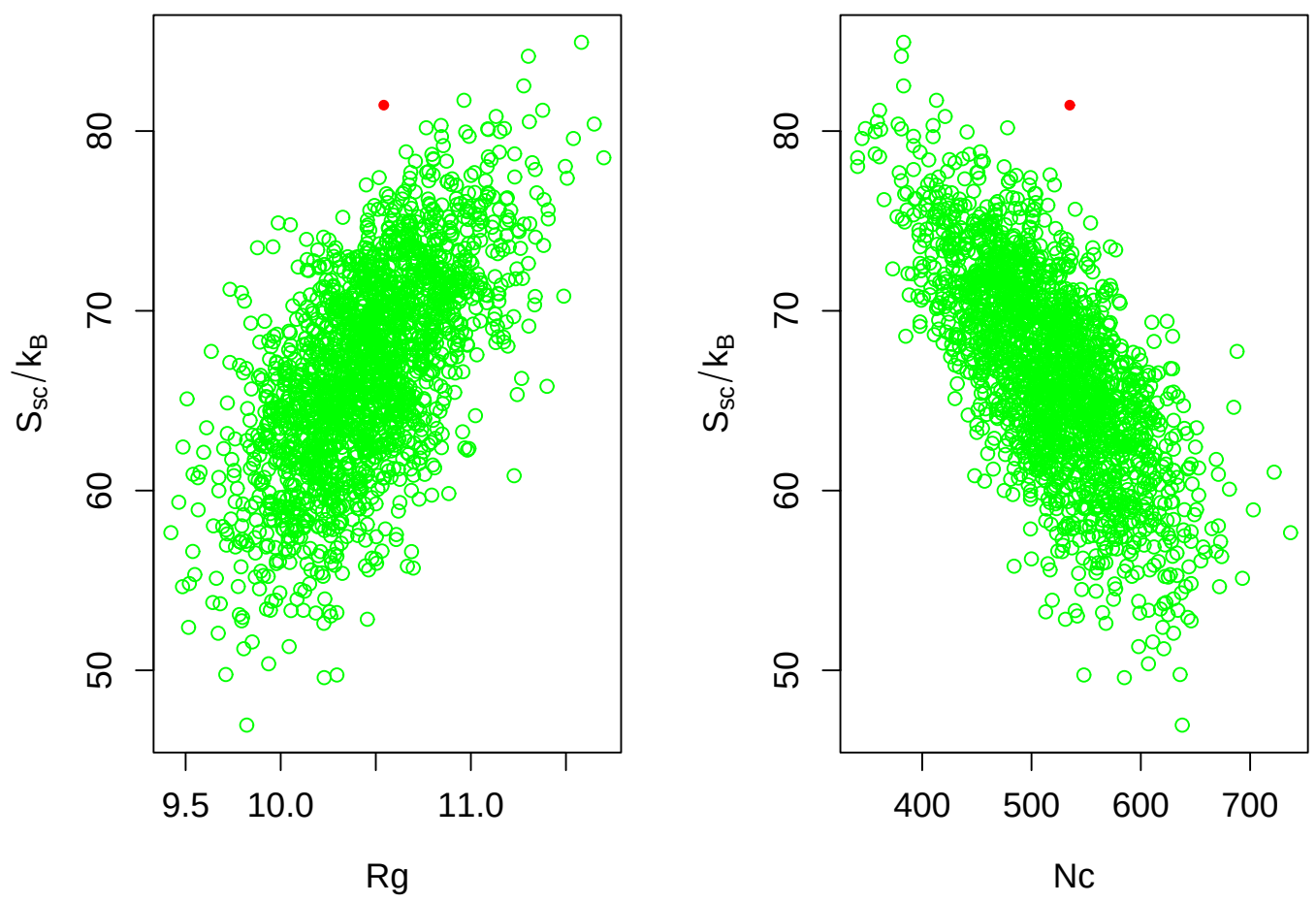

1dkt

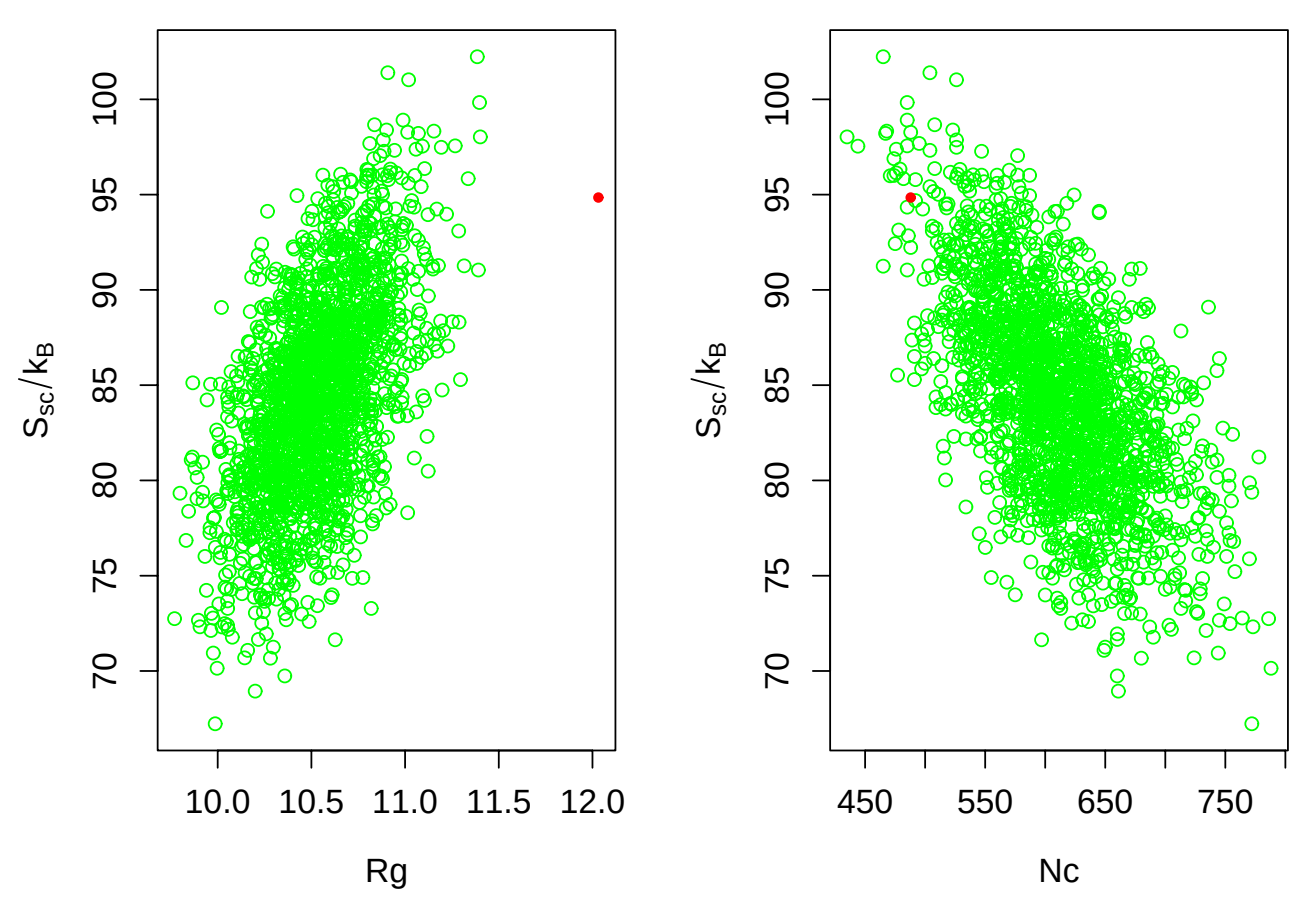

1fca

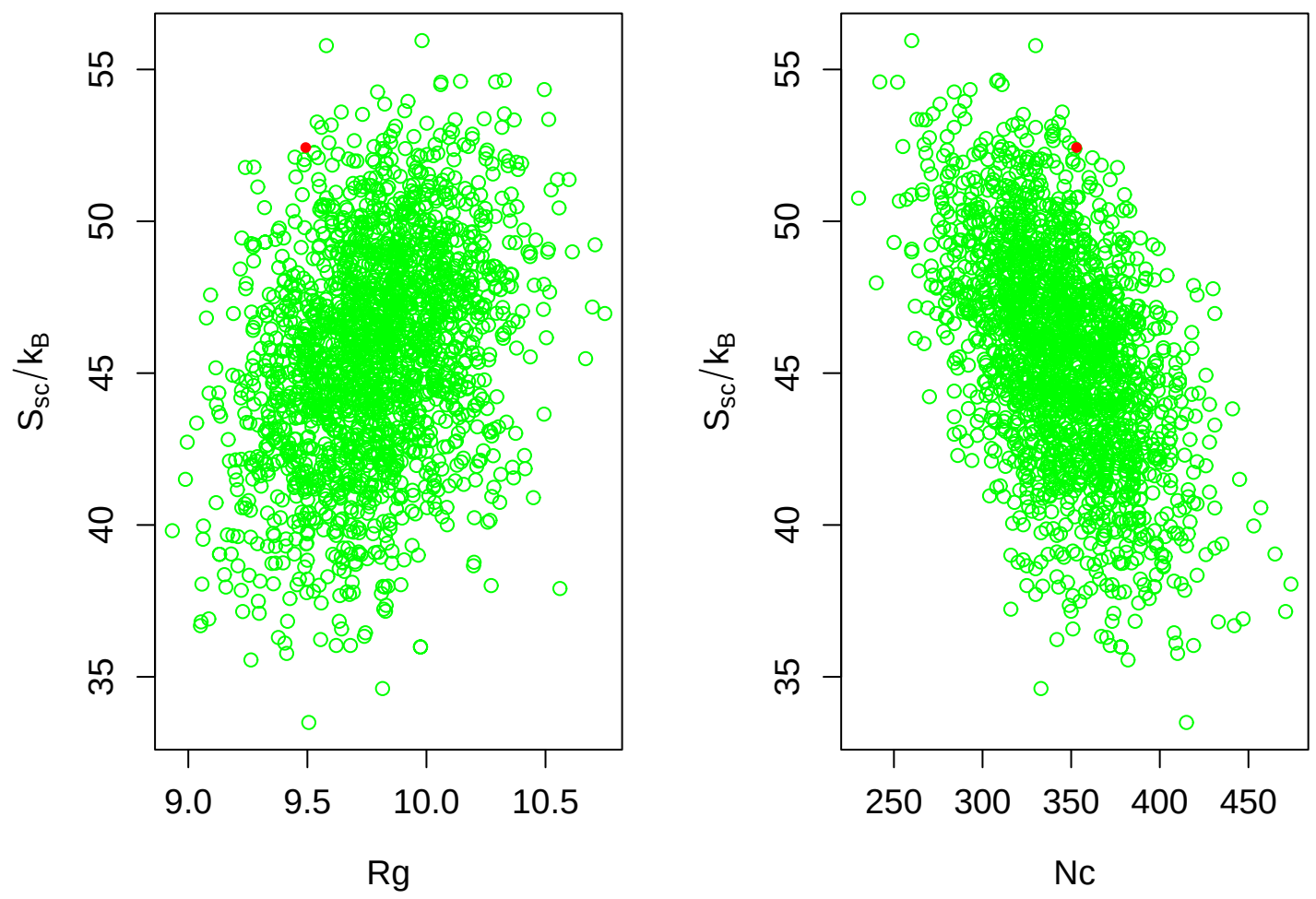

1nkl

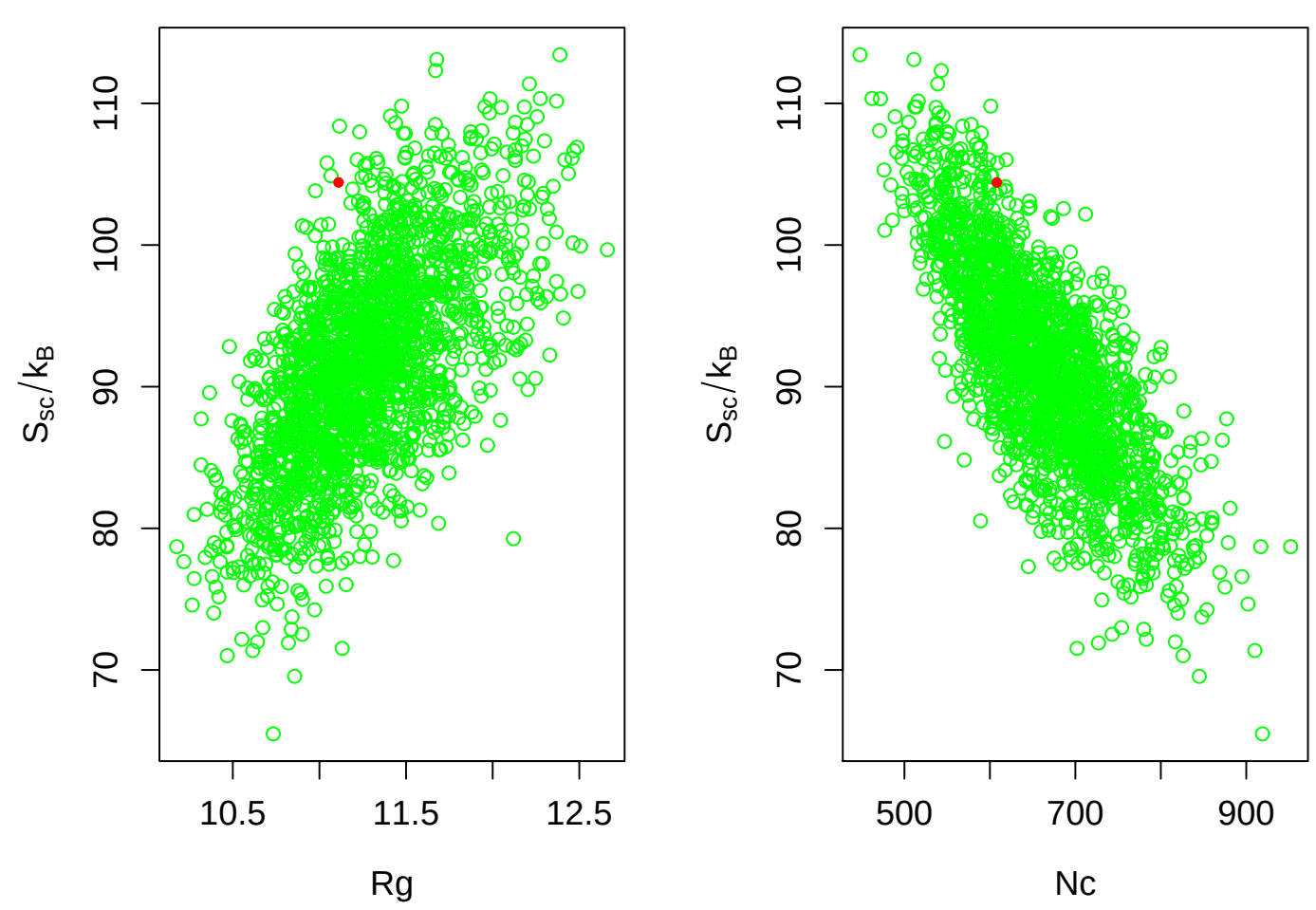

1pgb

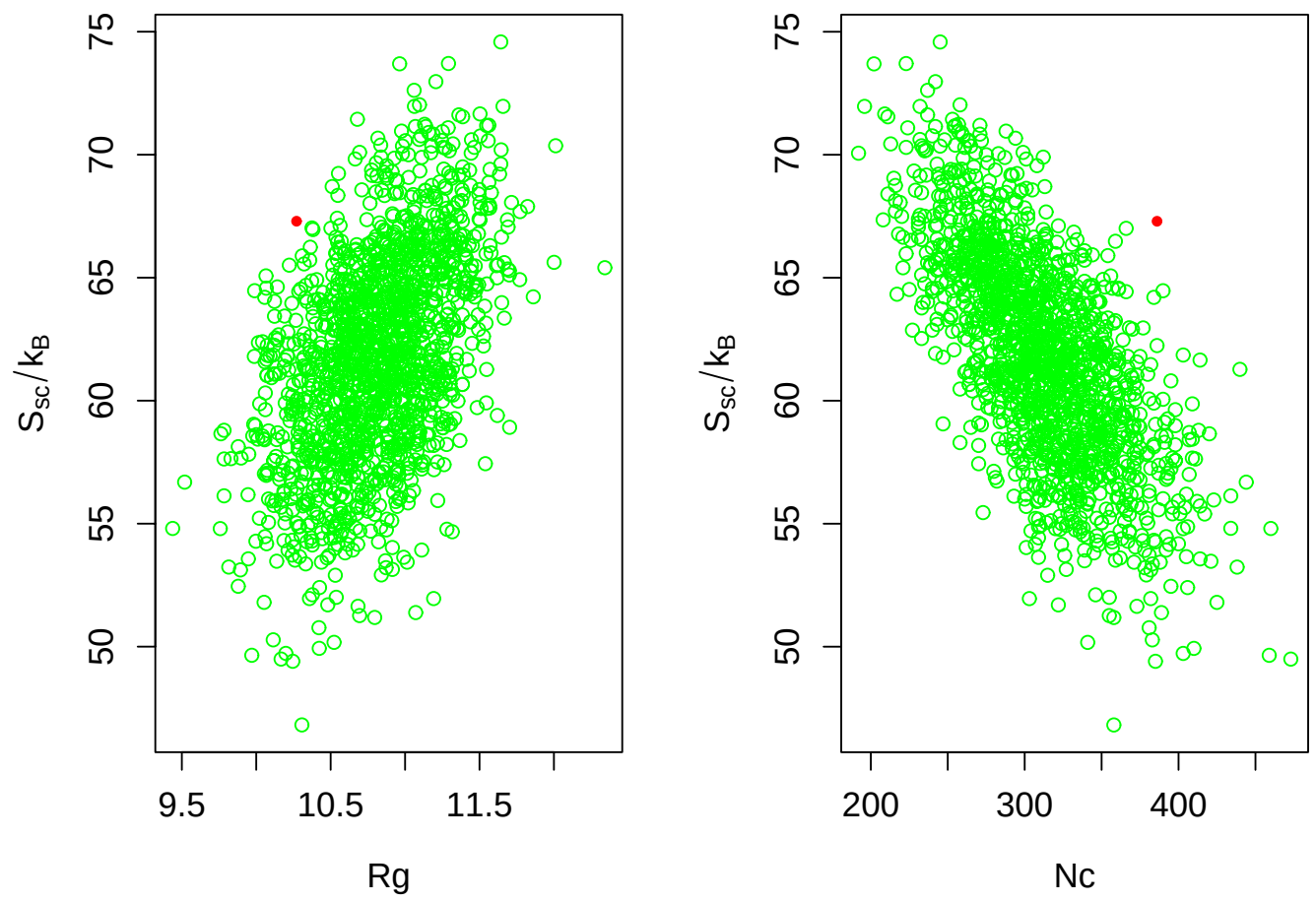

5. lmds

1ctf

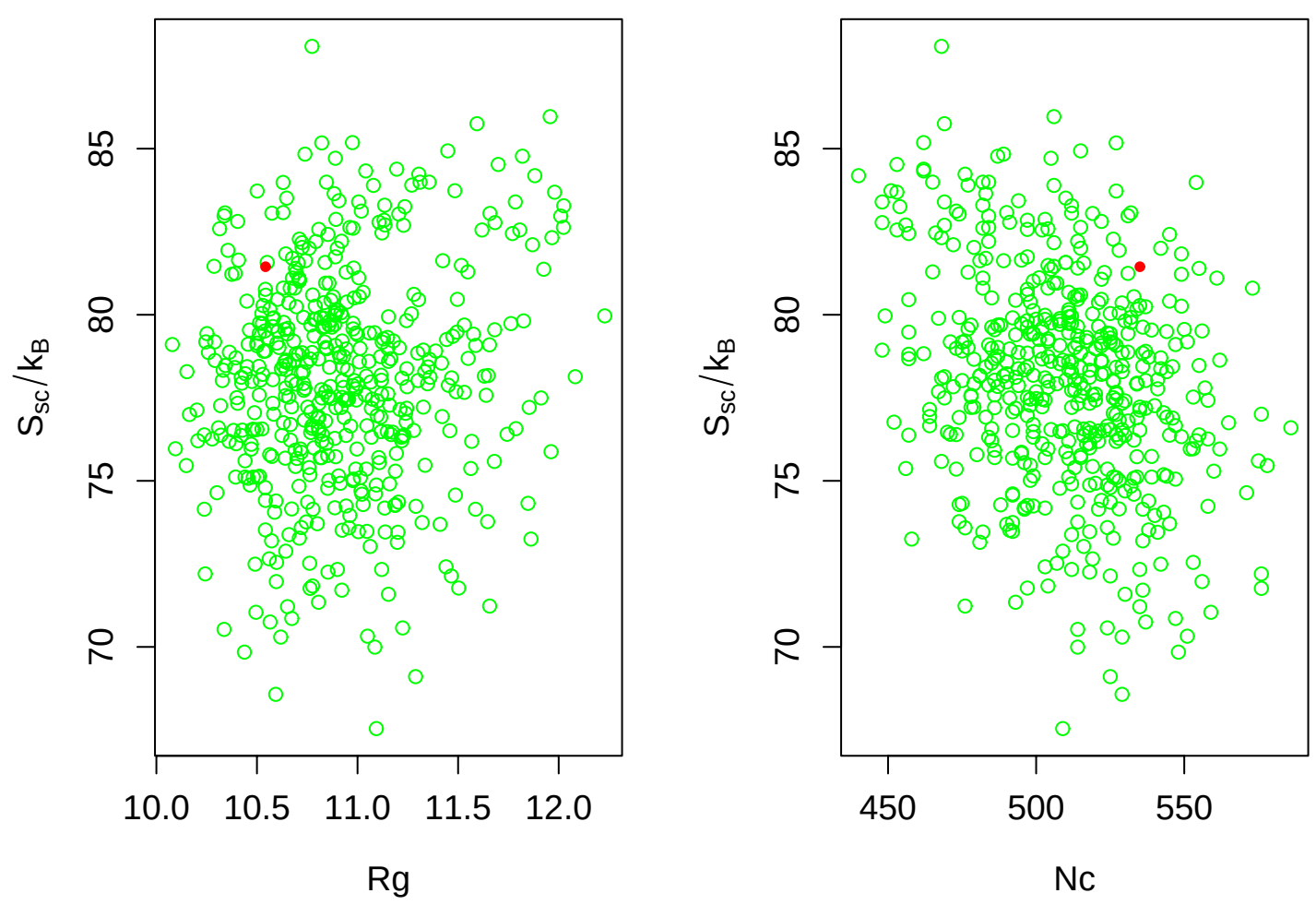

1fc2 (Protein interacting with another protein)

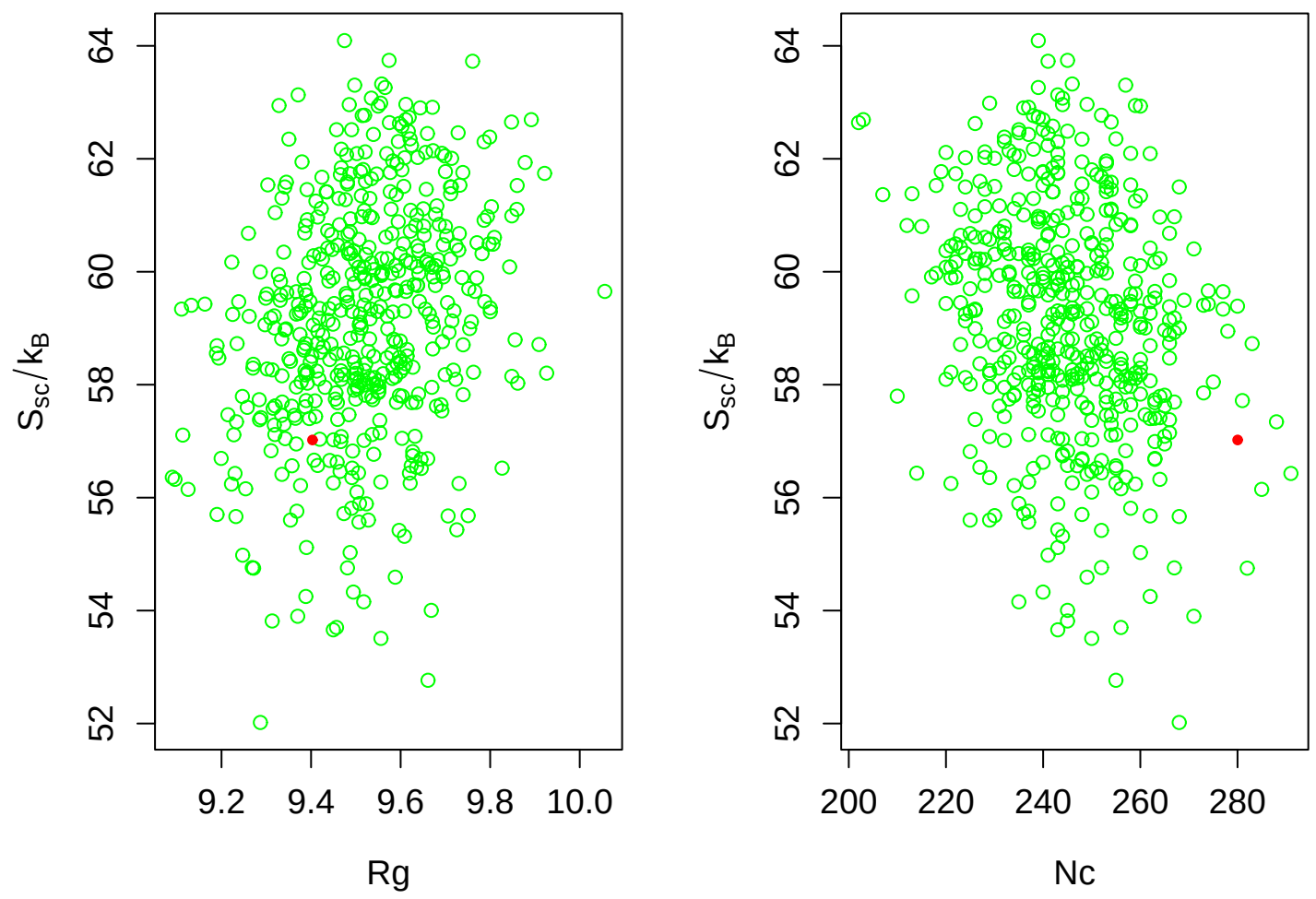

1igd

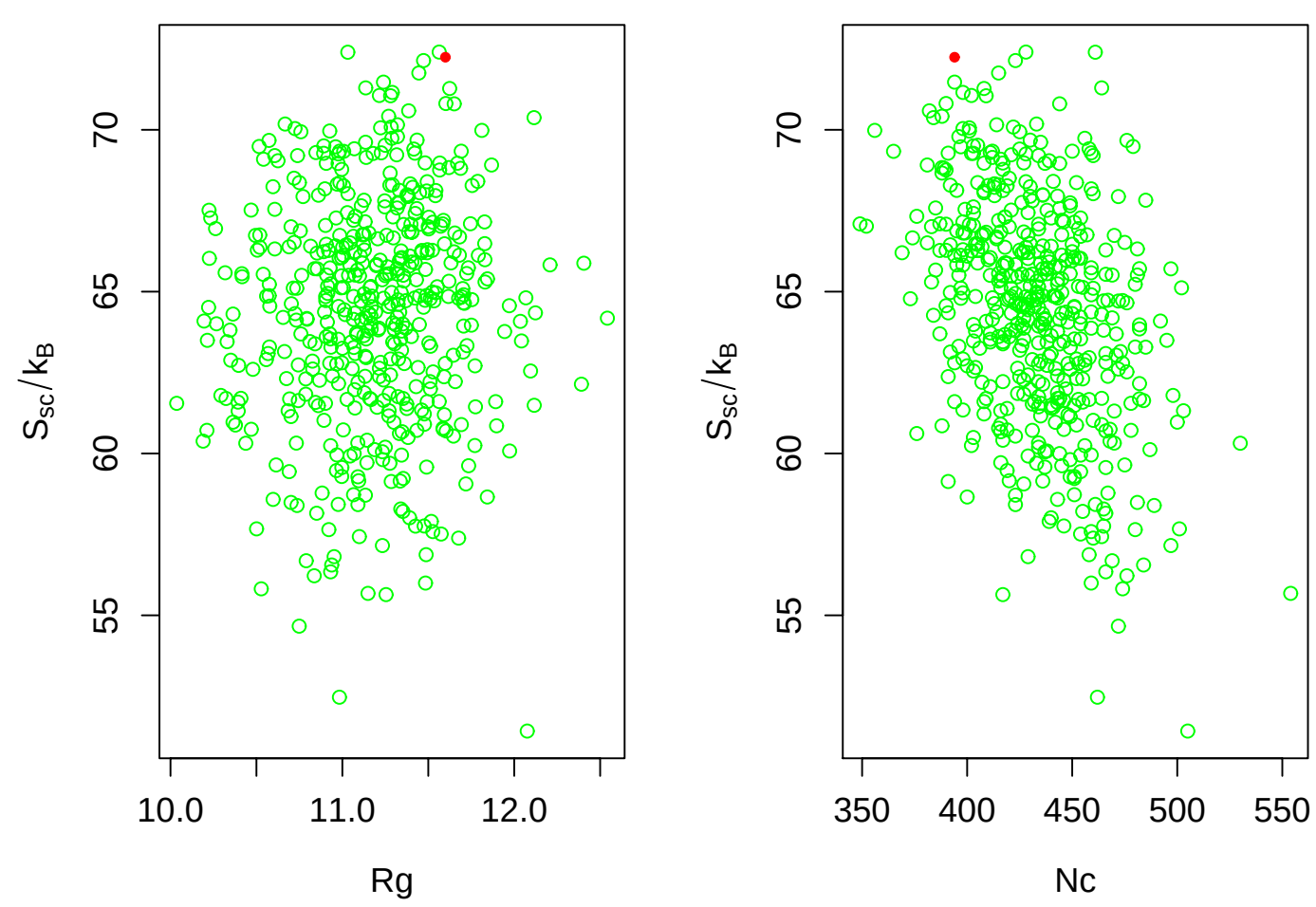

1shf-A

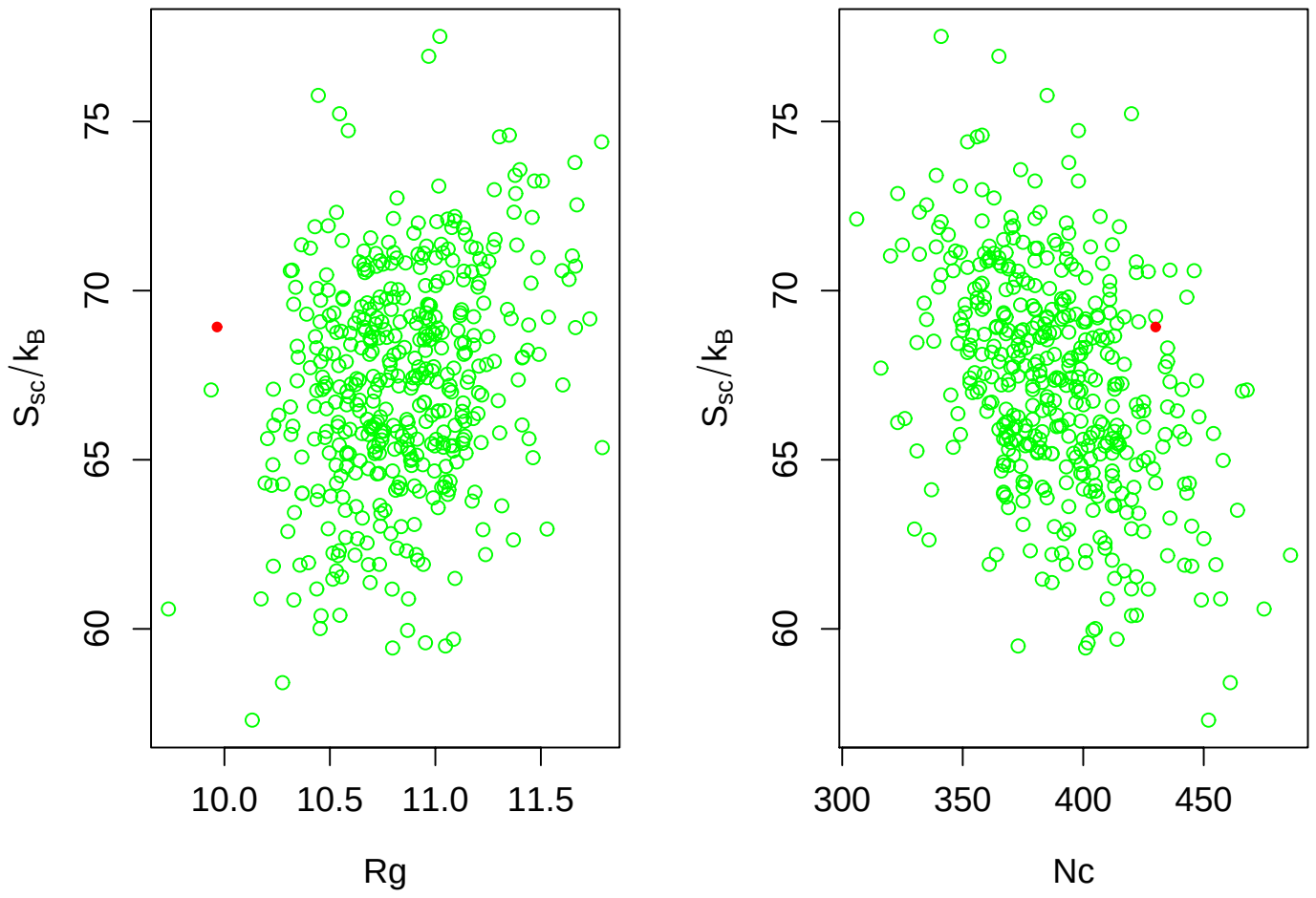

2cro

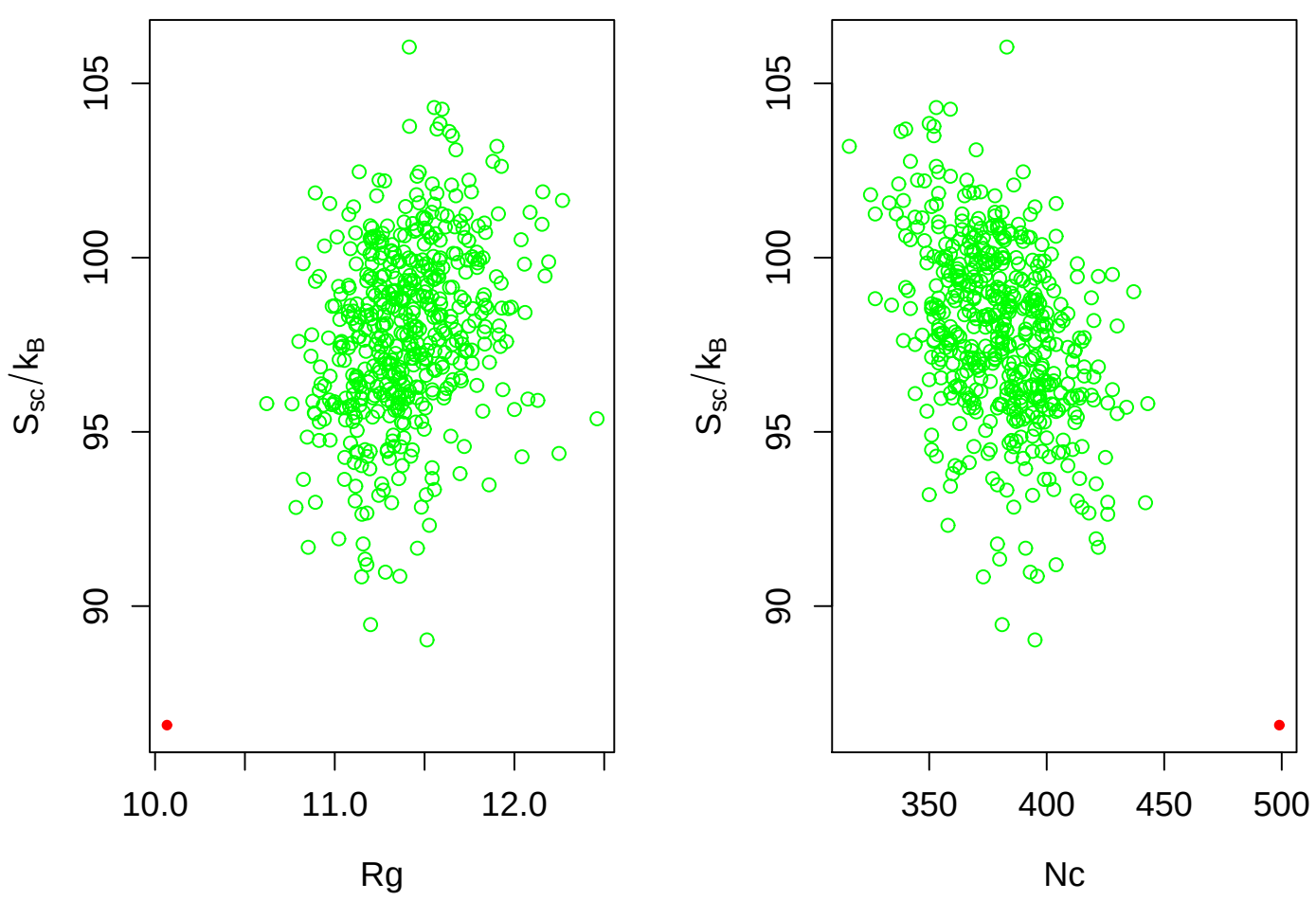

2ovo

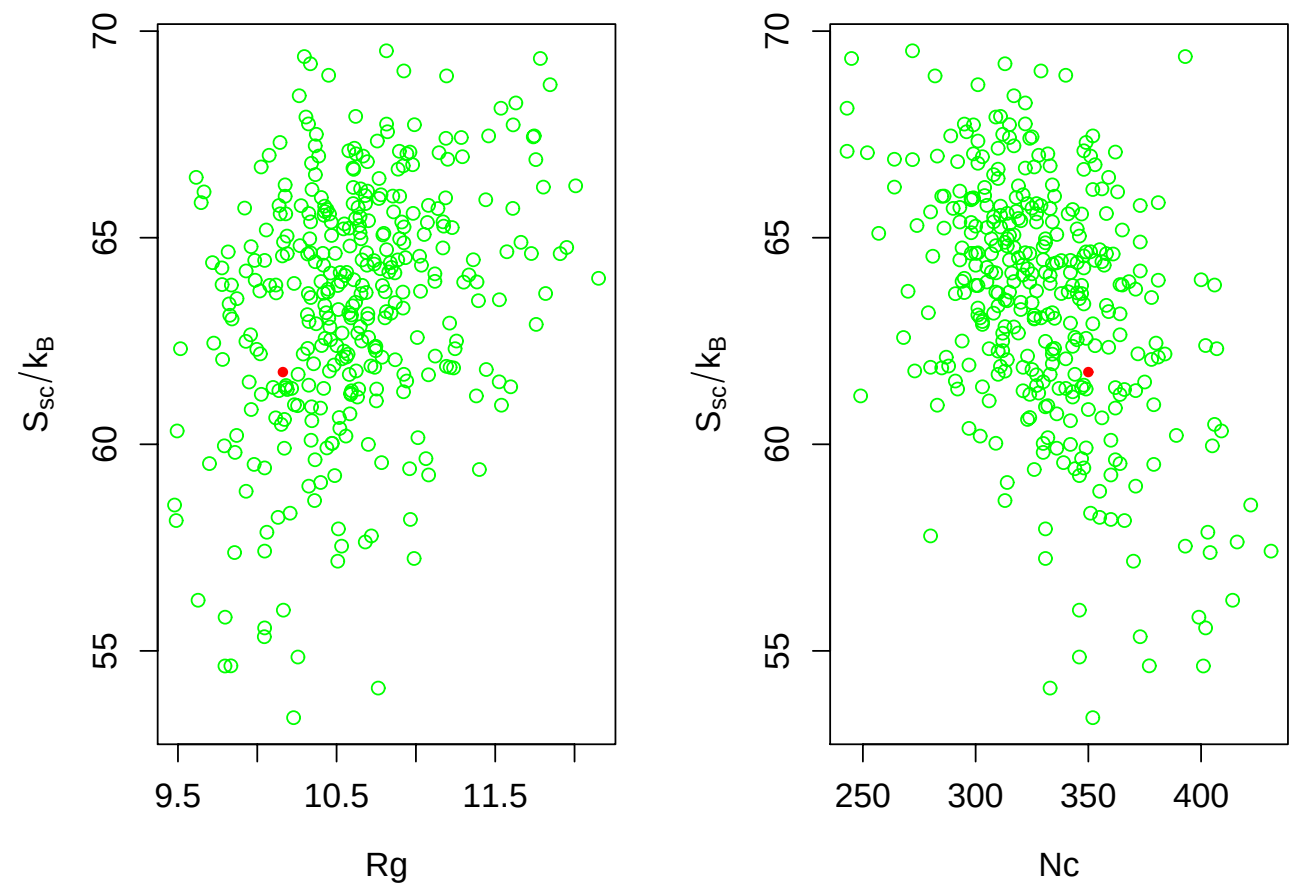

4pti (Protein with disulfide bonds)

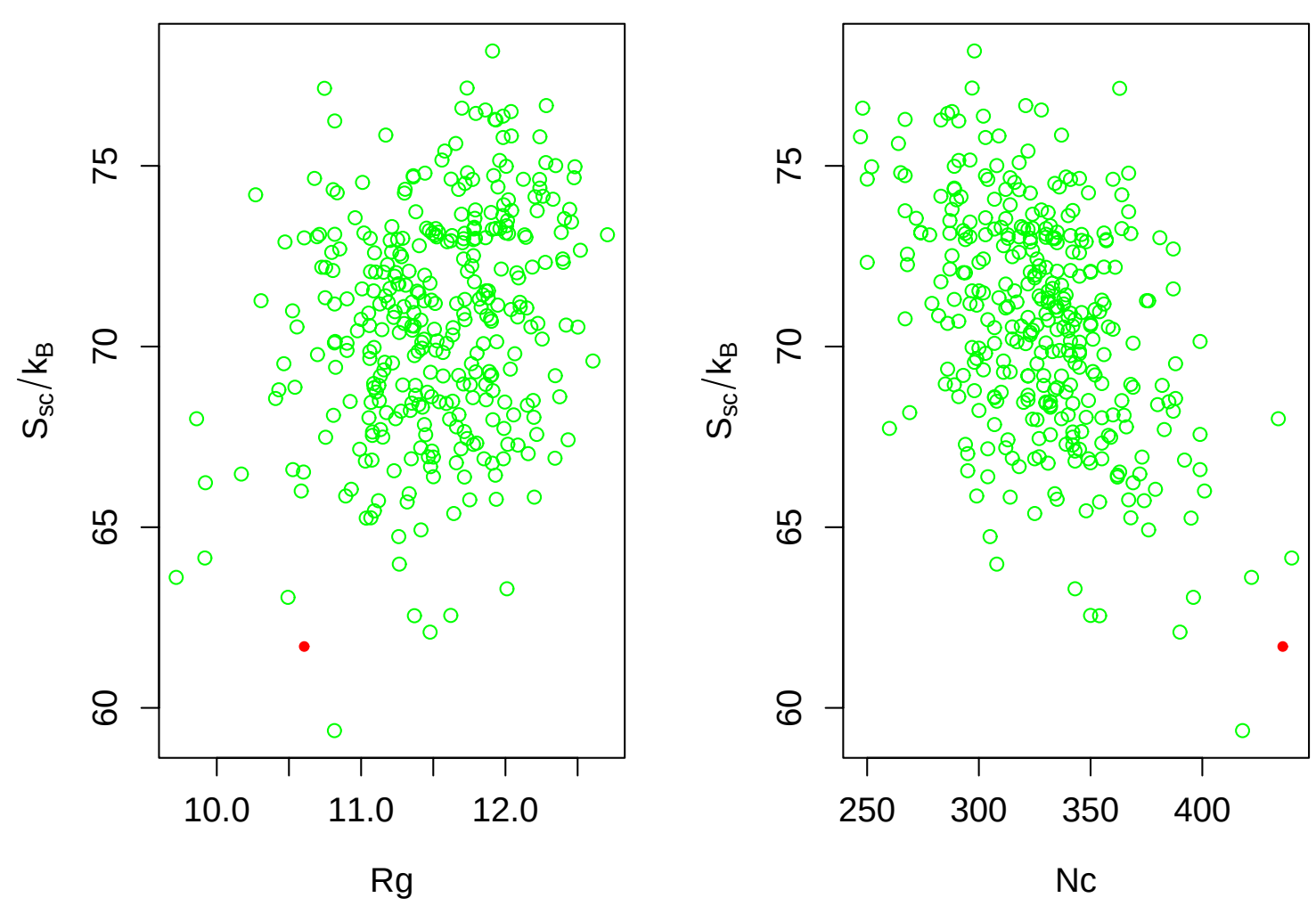

II. Side-chain entropy versus number of contacts for X-ray structures and their decoys of protein-protein complexes generated by Rosetta program. Native complexes are represented by black dot and decoys empty squares. For some proteins (1bvk, 1cse, 1nmb, 2btf, 3hhr), except native complex, all decoy structures have no self-avoiding conformations. Their figures are not shown.

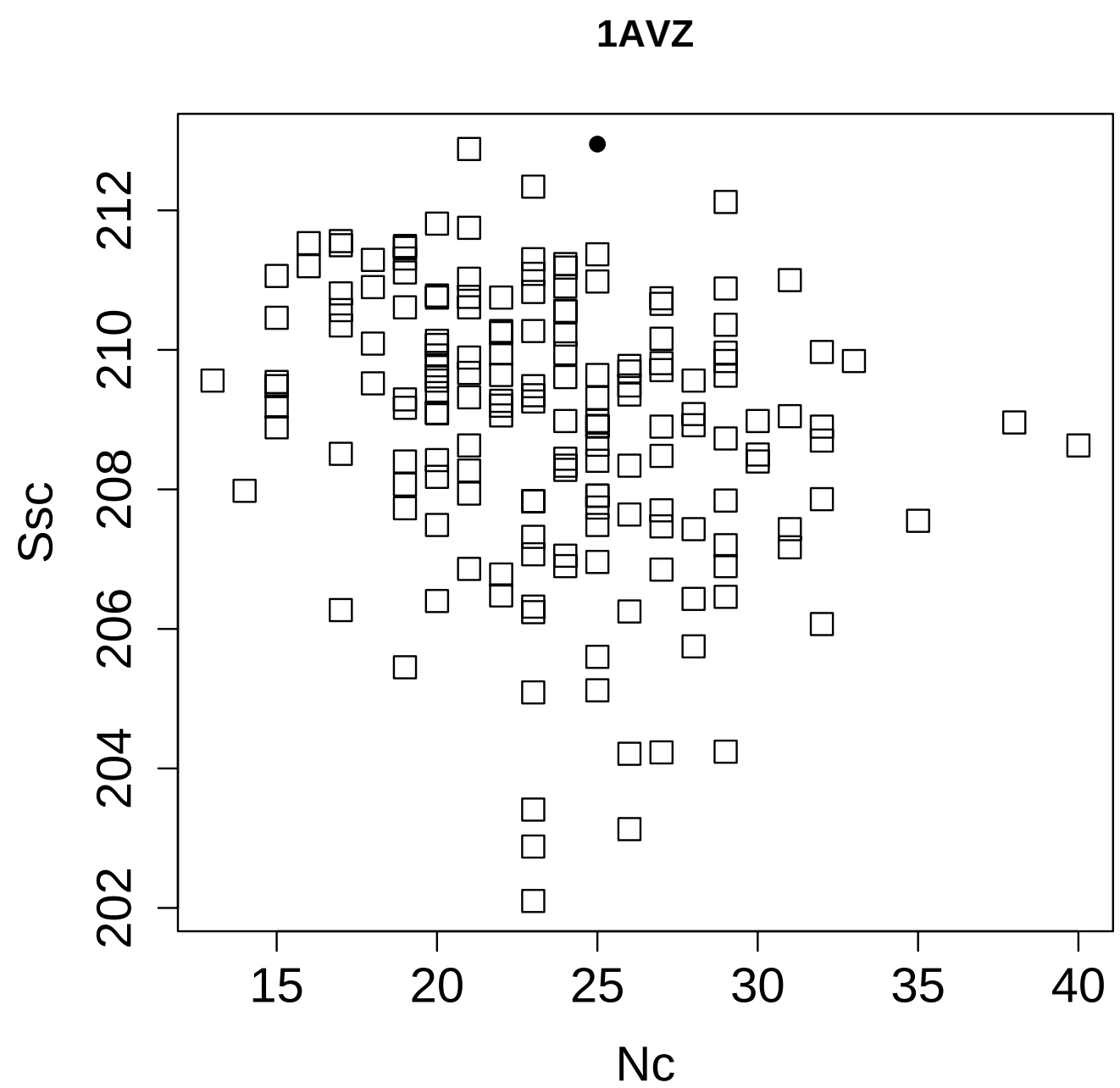
1AVZ
Ssc
Nc
202
204
206
208
210
212
15
20
25
30
35
40

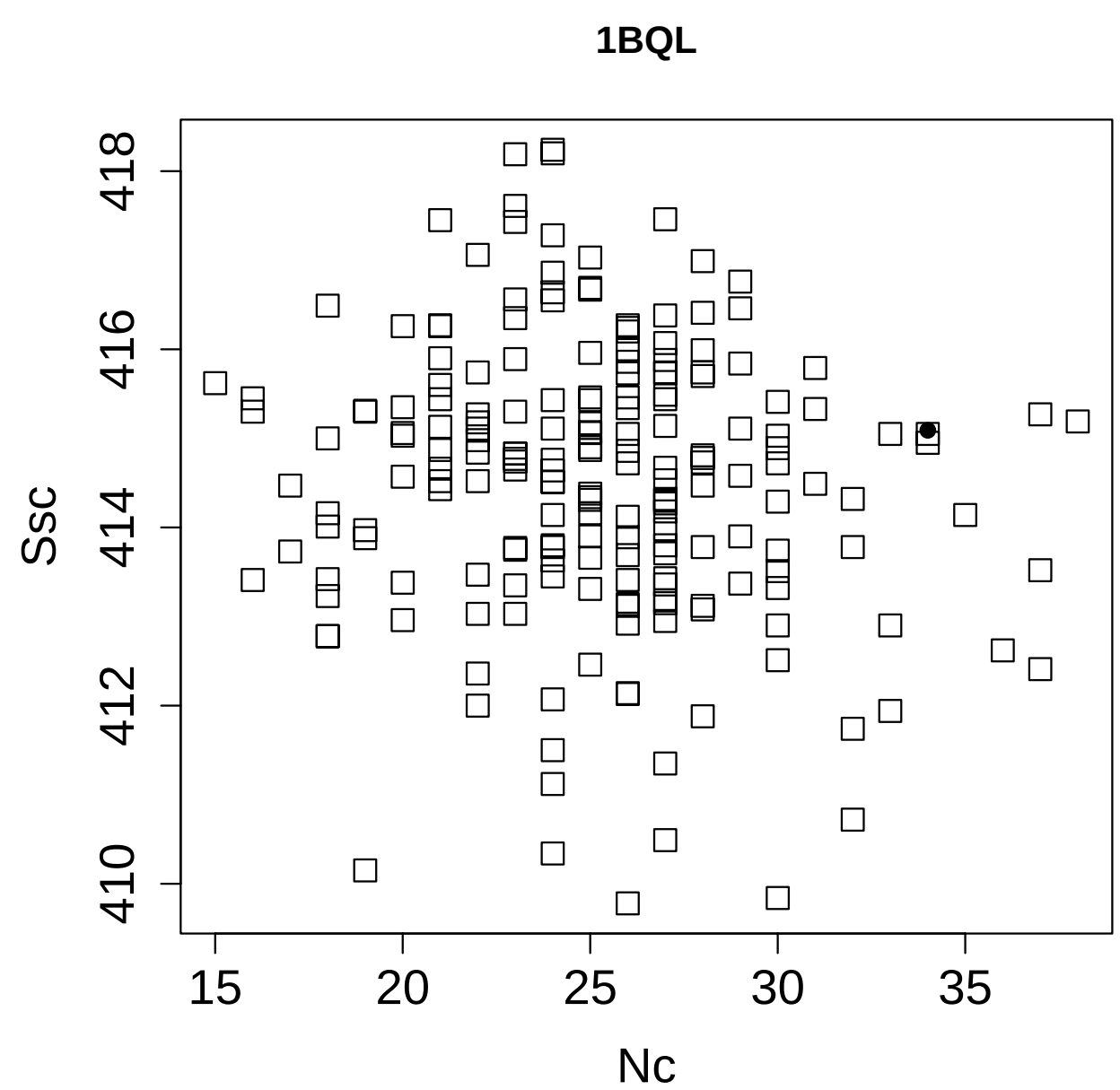
1BQL
Ssc
Nc
410
412
414
416
418
15
20
25
30
35

1BRC

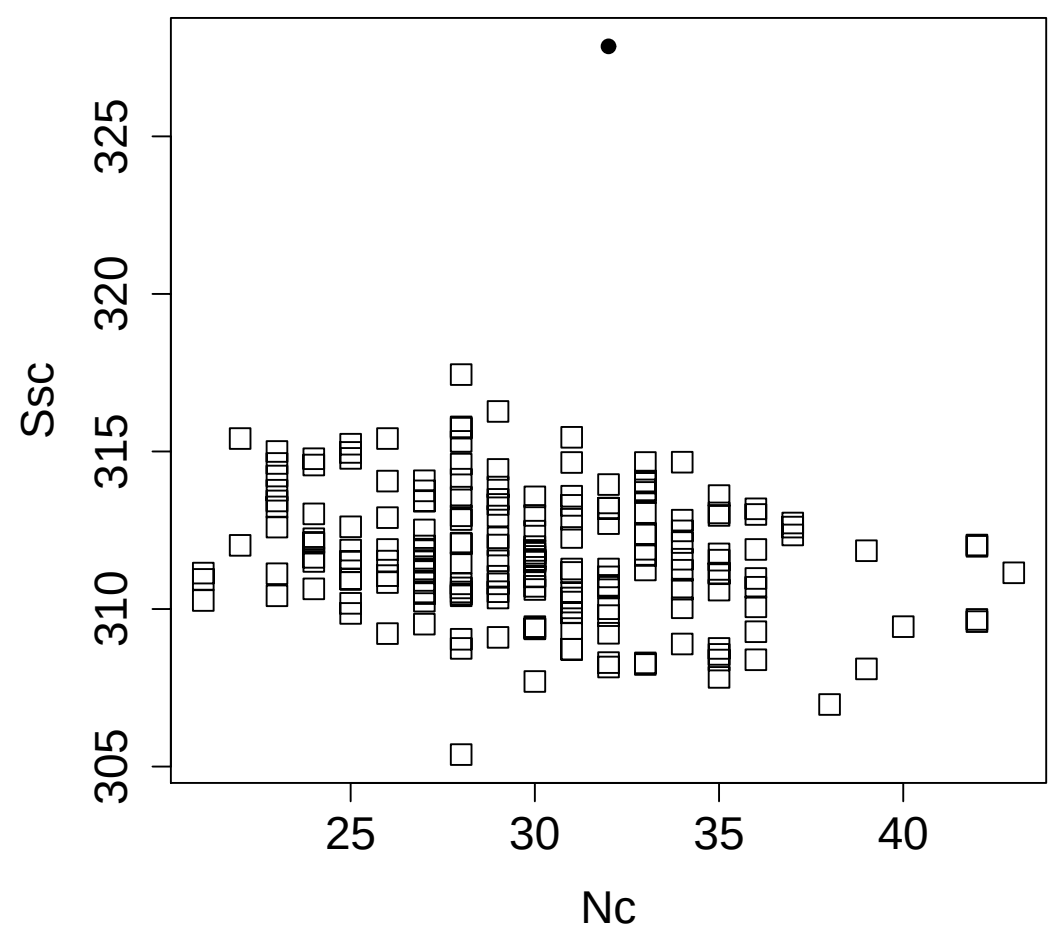

1BTH

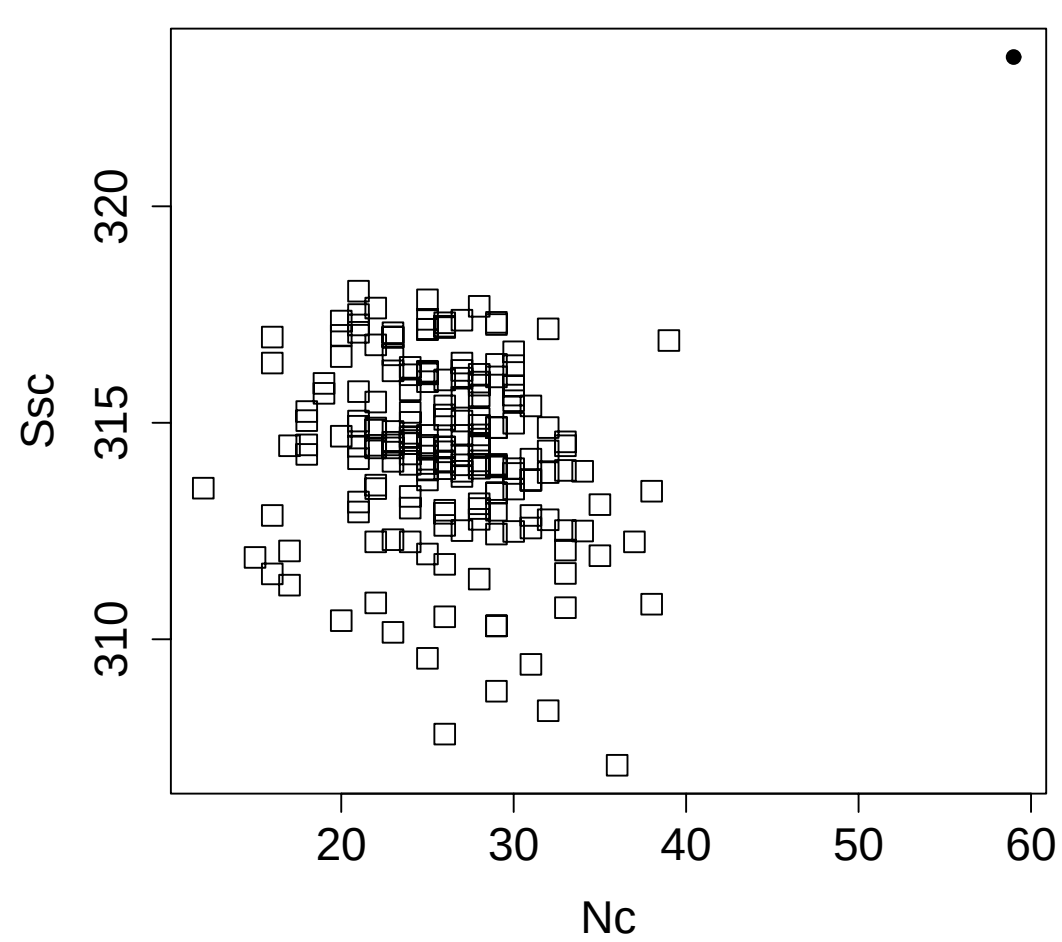

1DFJ

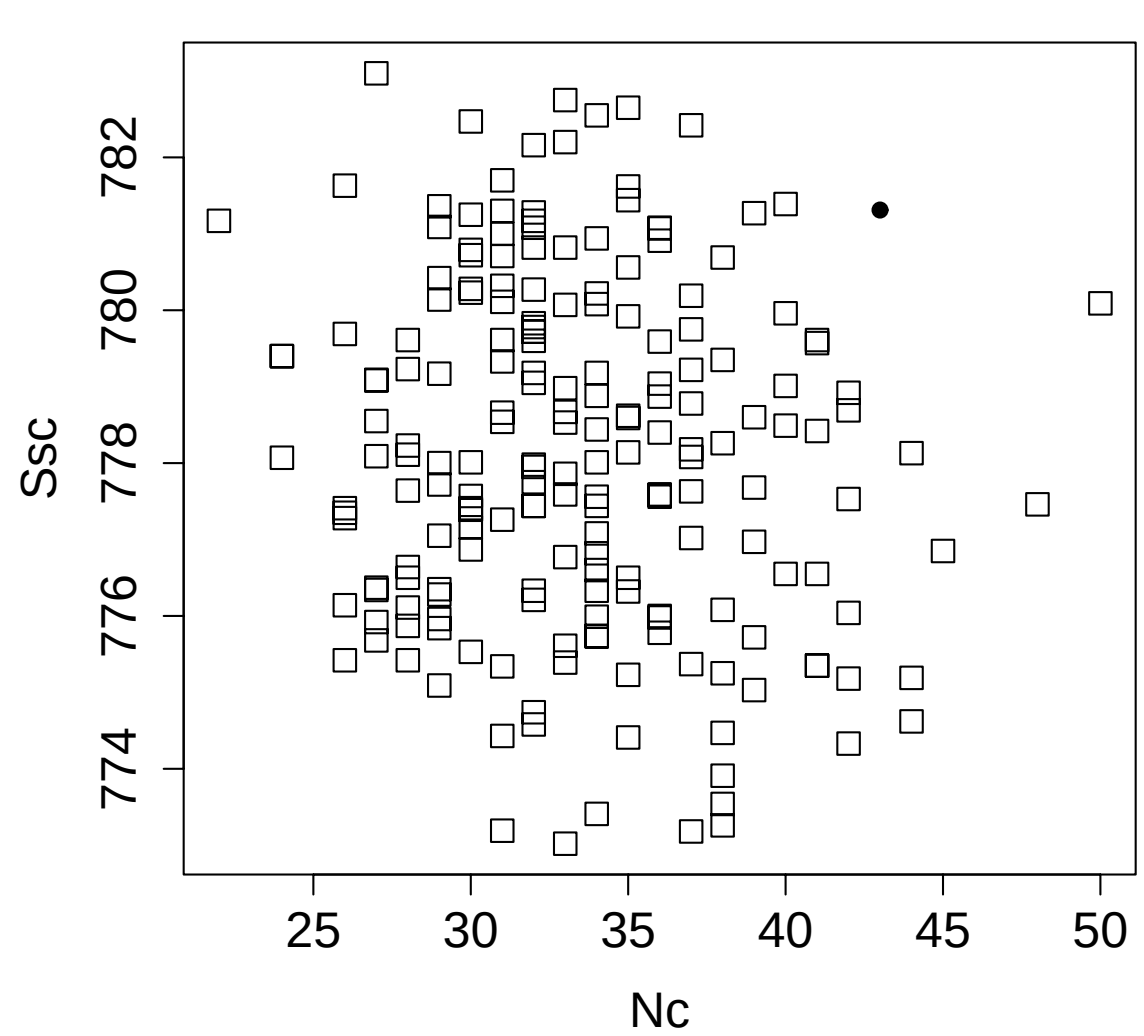

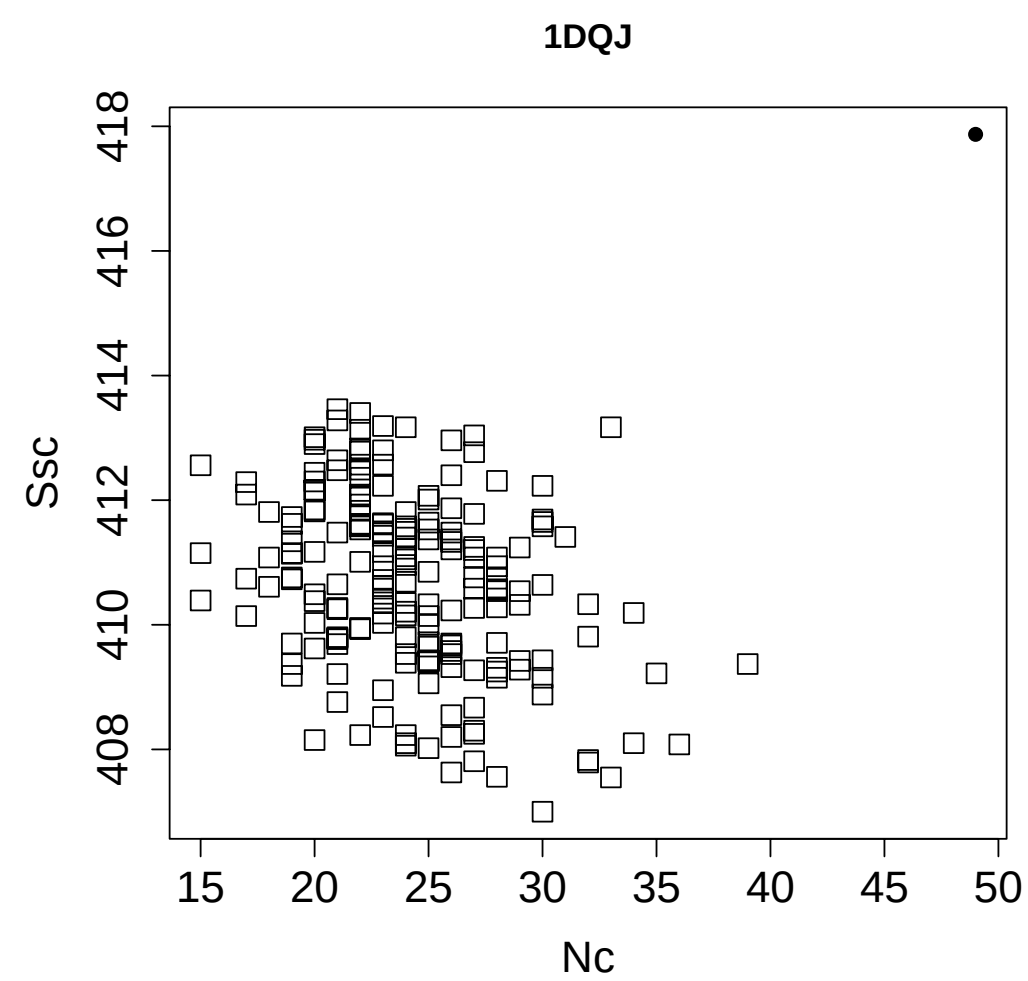
1DQJ
Ssc
Nc
408
410
412
414
416
418
15
20
25
30
35
40
45
50

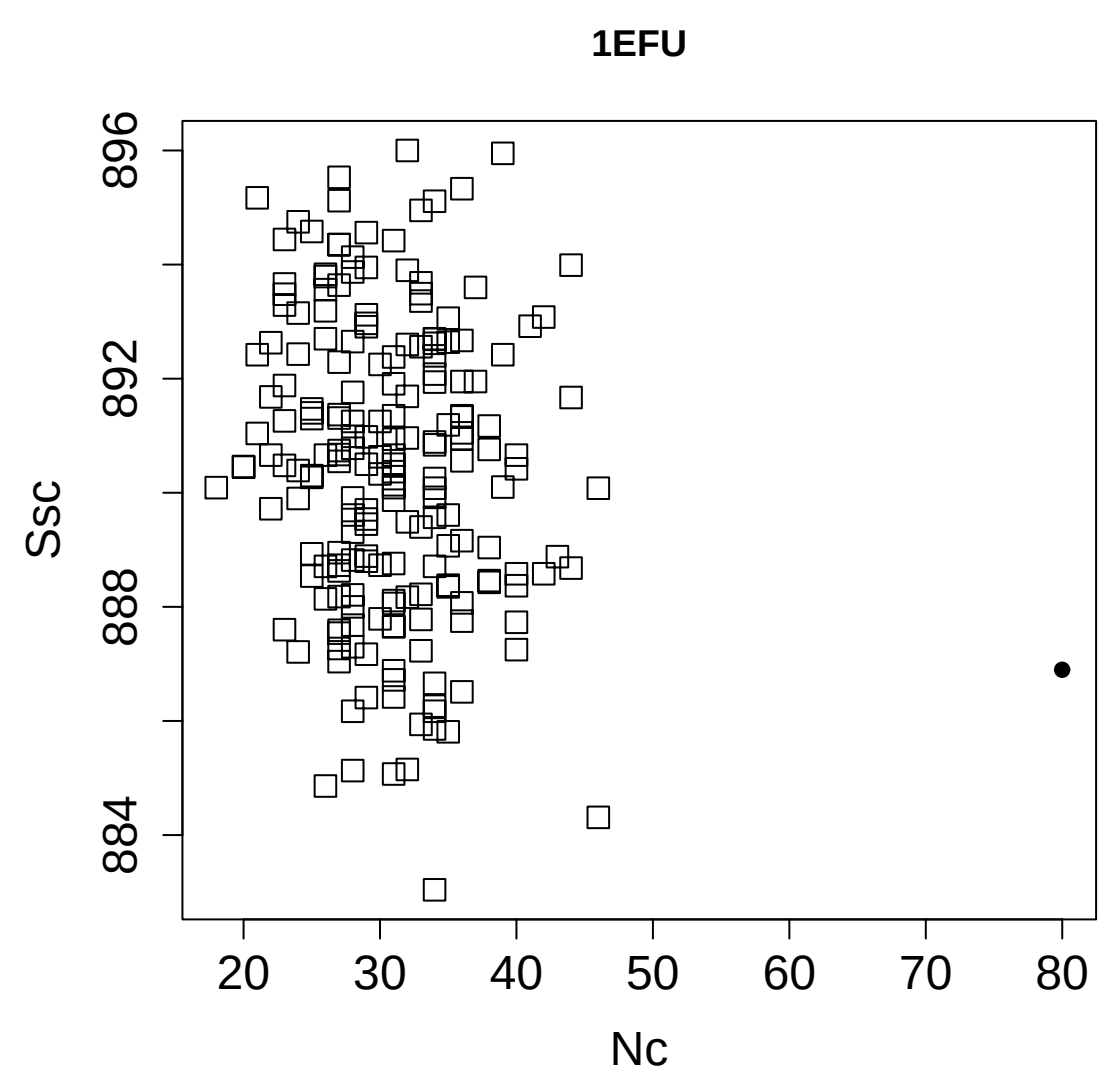
1EFU
Ssc
Nc
884
888
892
896
20
30
40
50
60
70
80

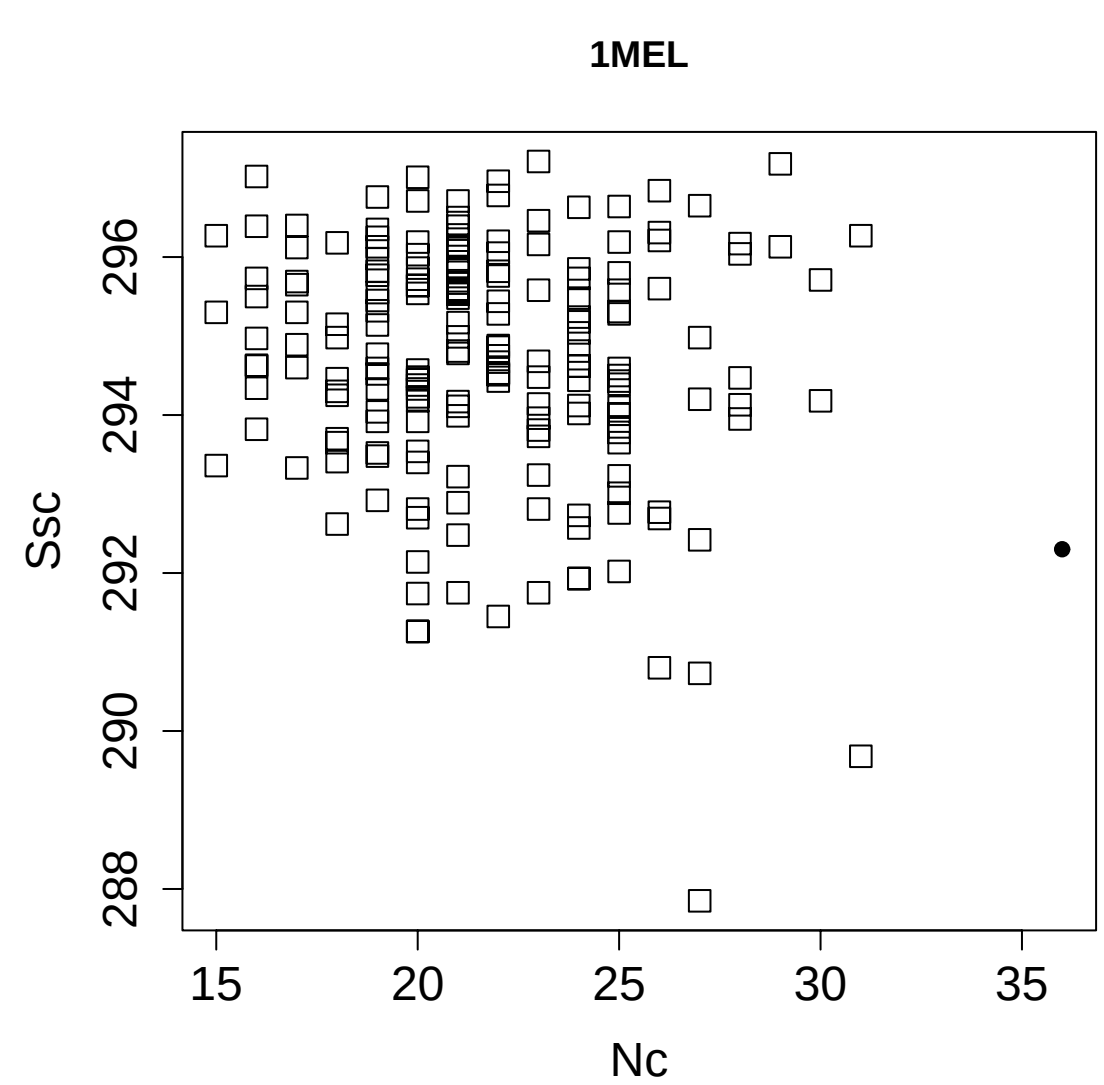
1MEL
Ssc
Nc
288
290
292
294
296
15
20
25
30
35

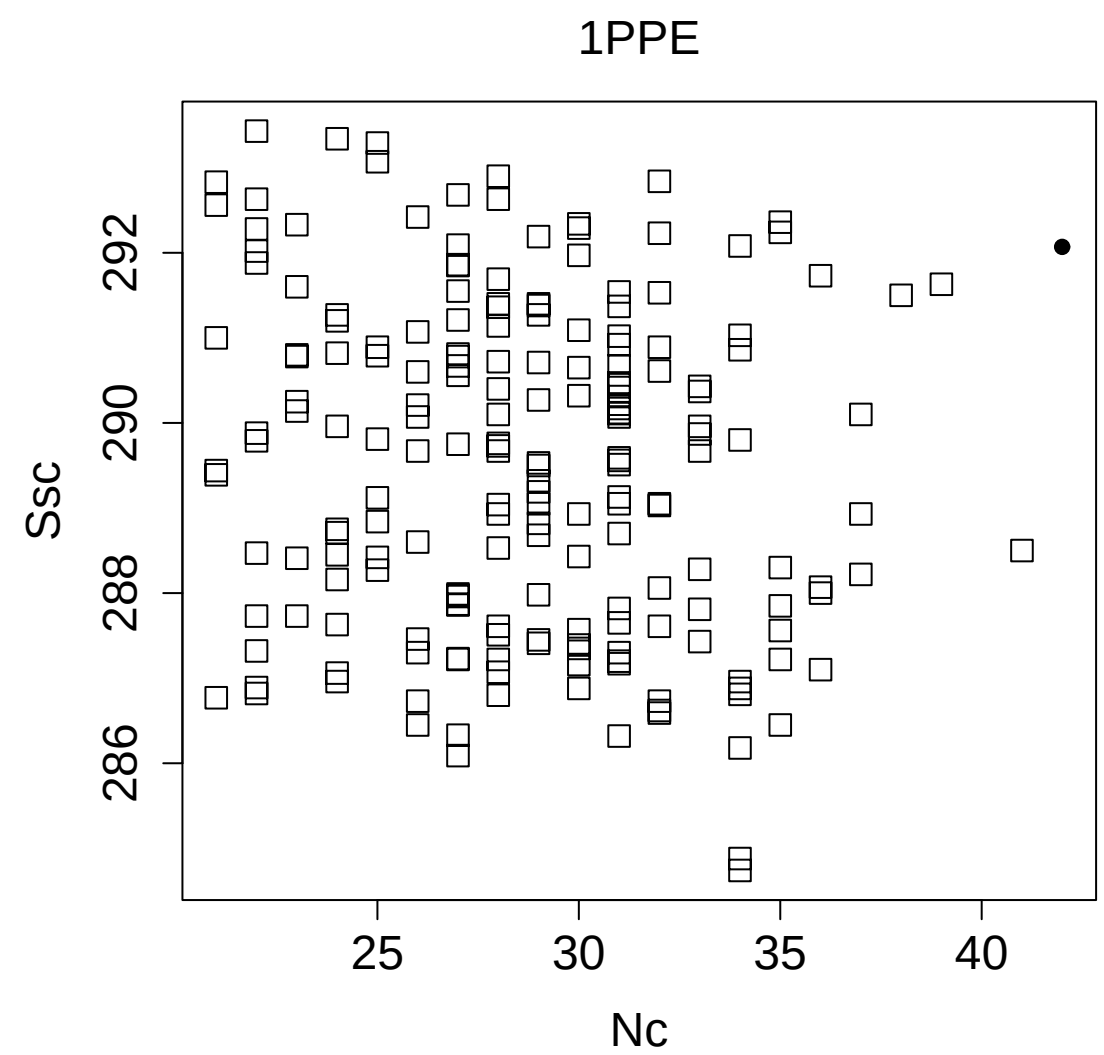
1PPE
Ssc
286
288
290
292
25
30
35
40
Nc

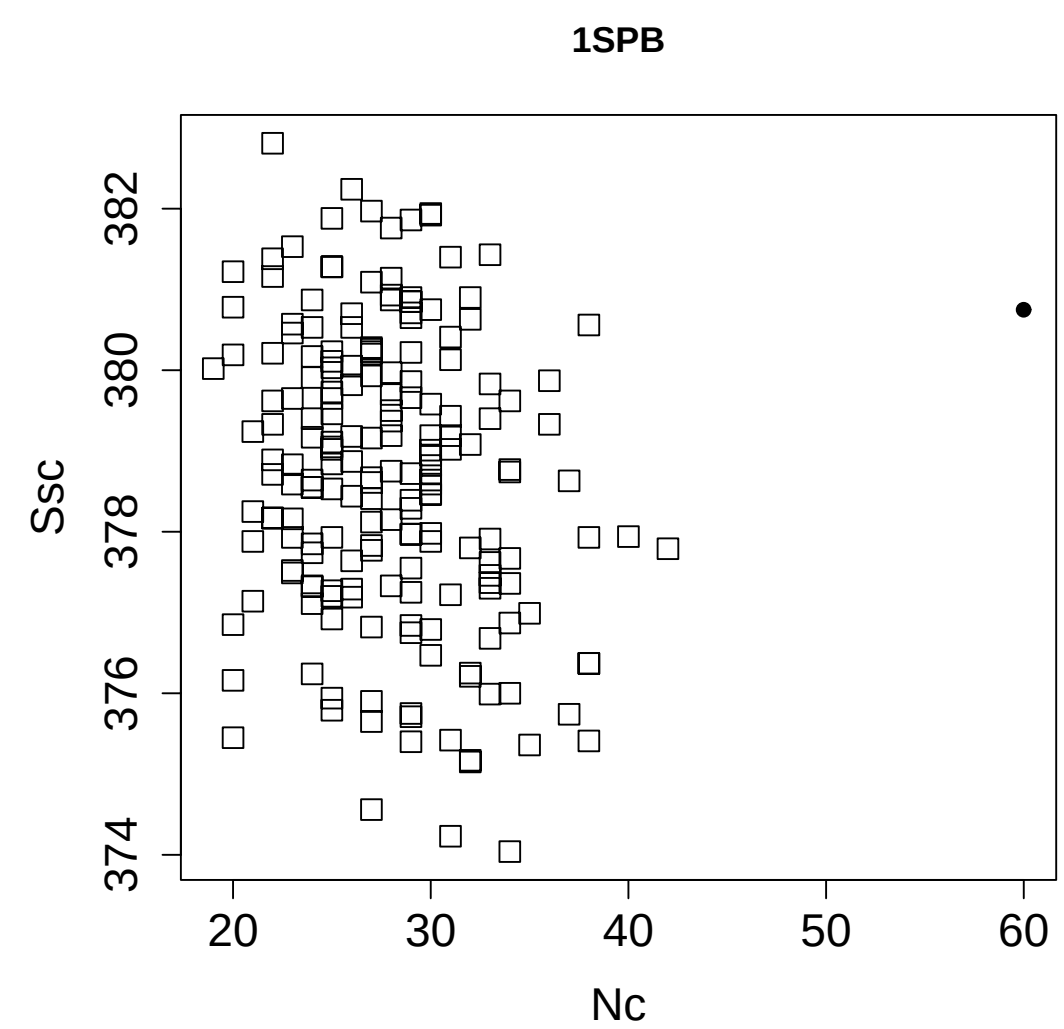
1SPB
Ssc
374
376
378
380
382
20
30
40
50
60
Nc

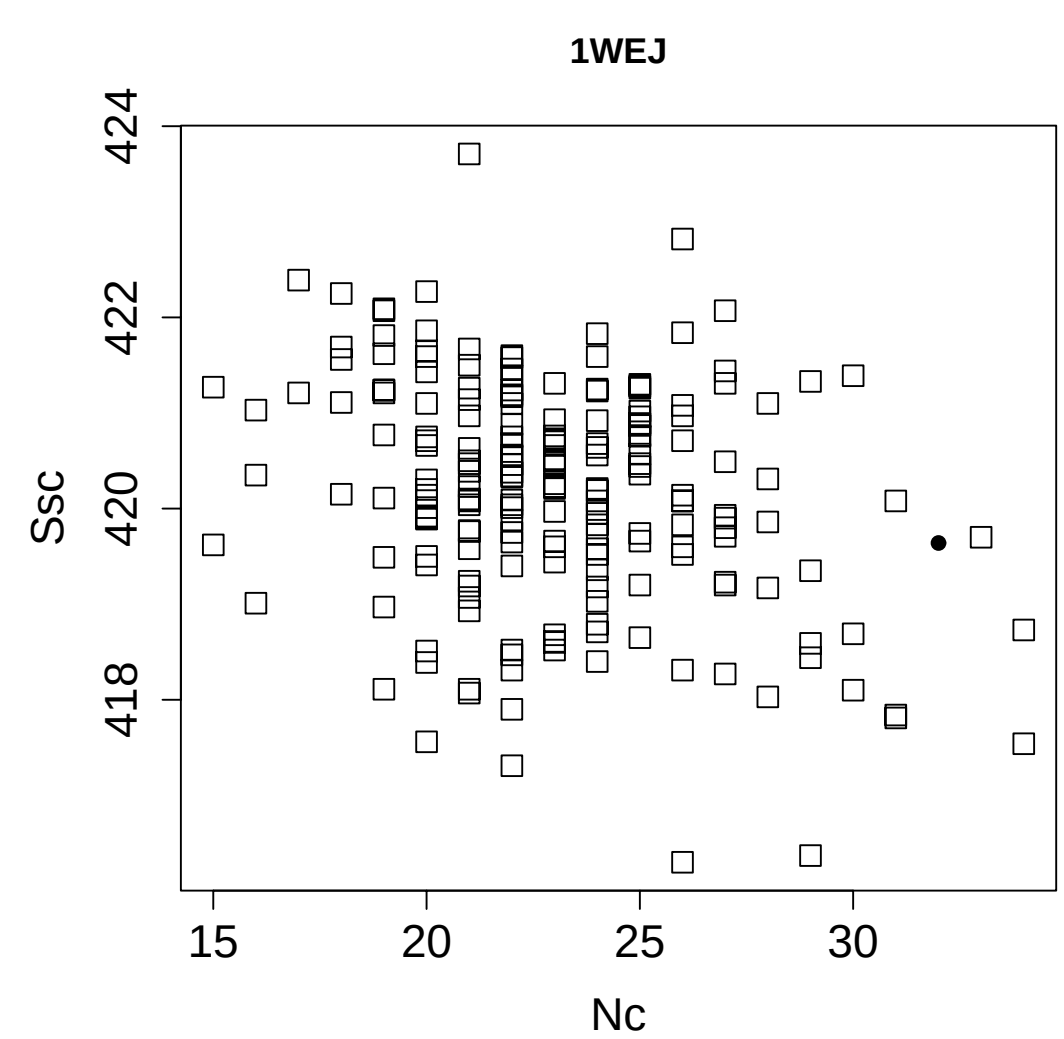
1WEJ
Ssc
418
420
422
424
15
20
25
30
Nc

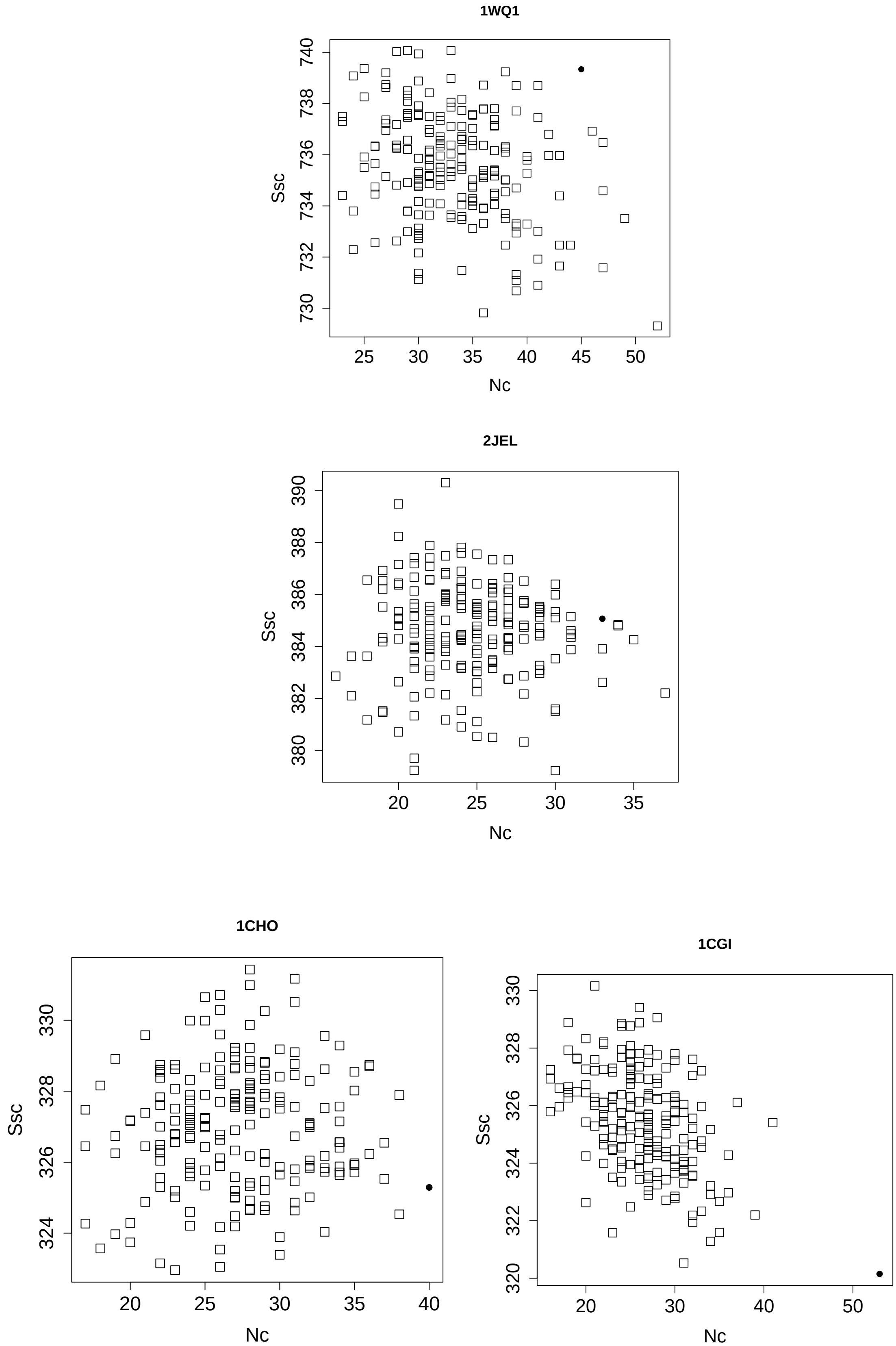
1WQ1
Ssc
740
738
736
734
732
730
25
30
35
40
45
50
Nc
2JEL
Ssc
390
388
386
384
382
380
20
25
30
35
Nc
1CHO
Ssc
330
328
326
324
20
25
30
35
40
Nc
1CGI
Ssc
330
328
326
324
322
320
20
30
40
50
Nc

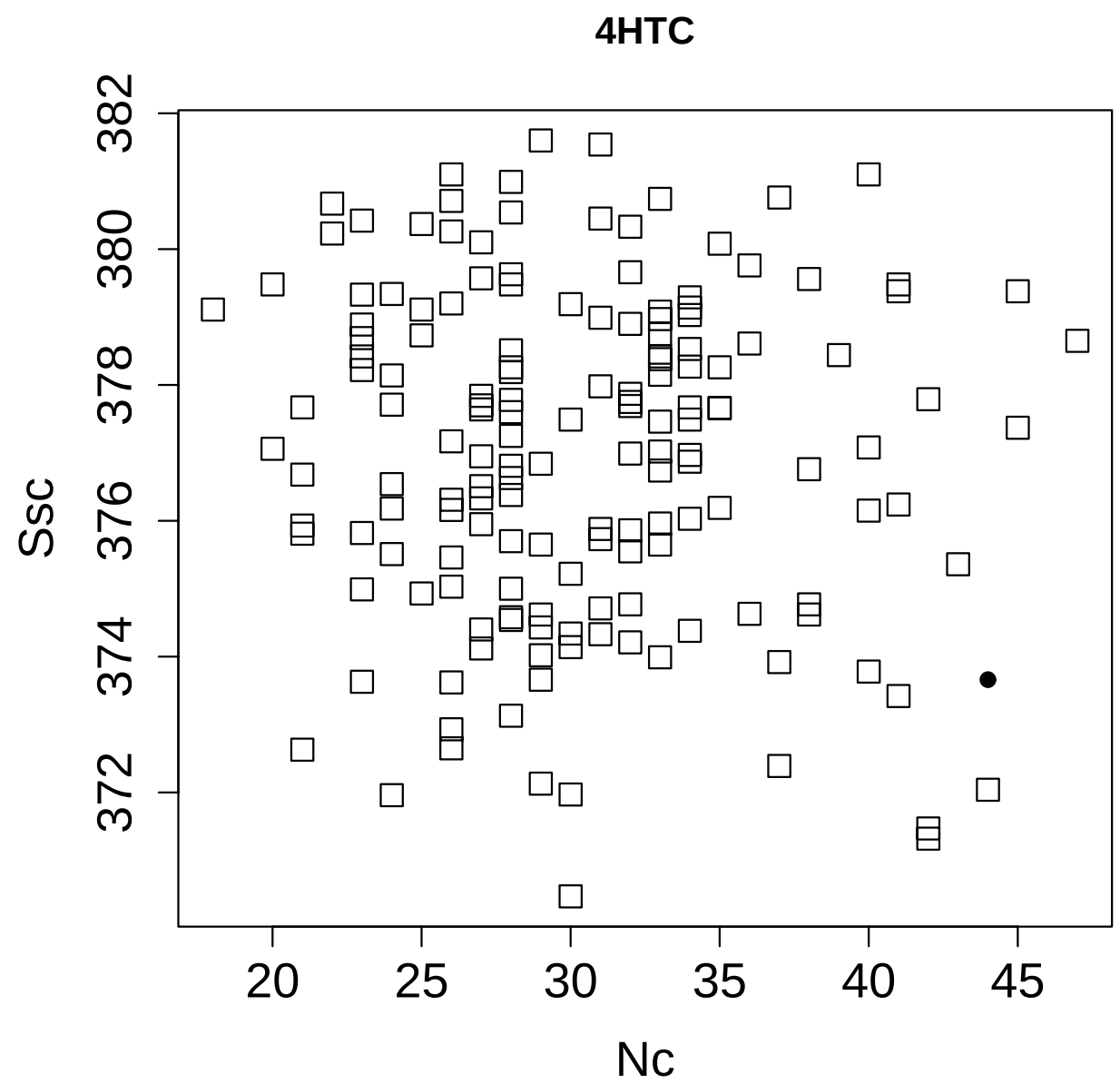
4HTC
Ssc
Nc
382
380
378
376
374
372
20
25
30
35
40
45

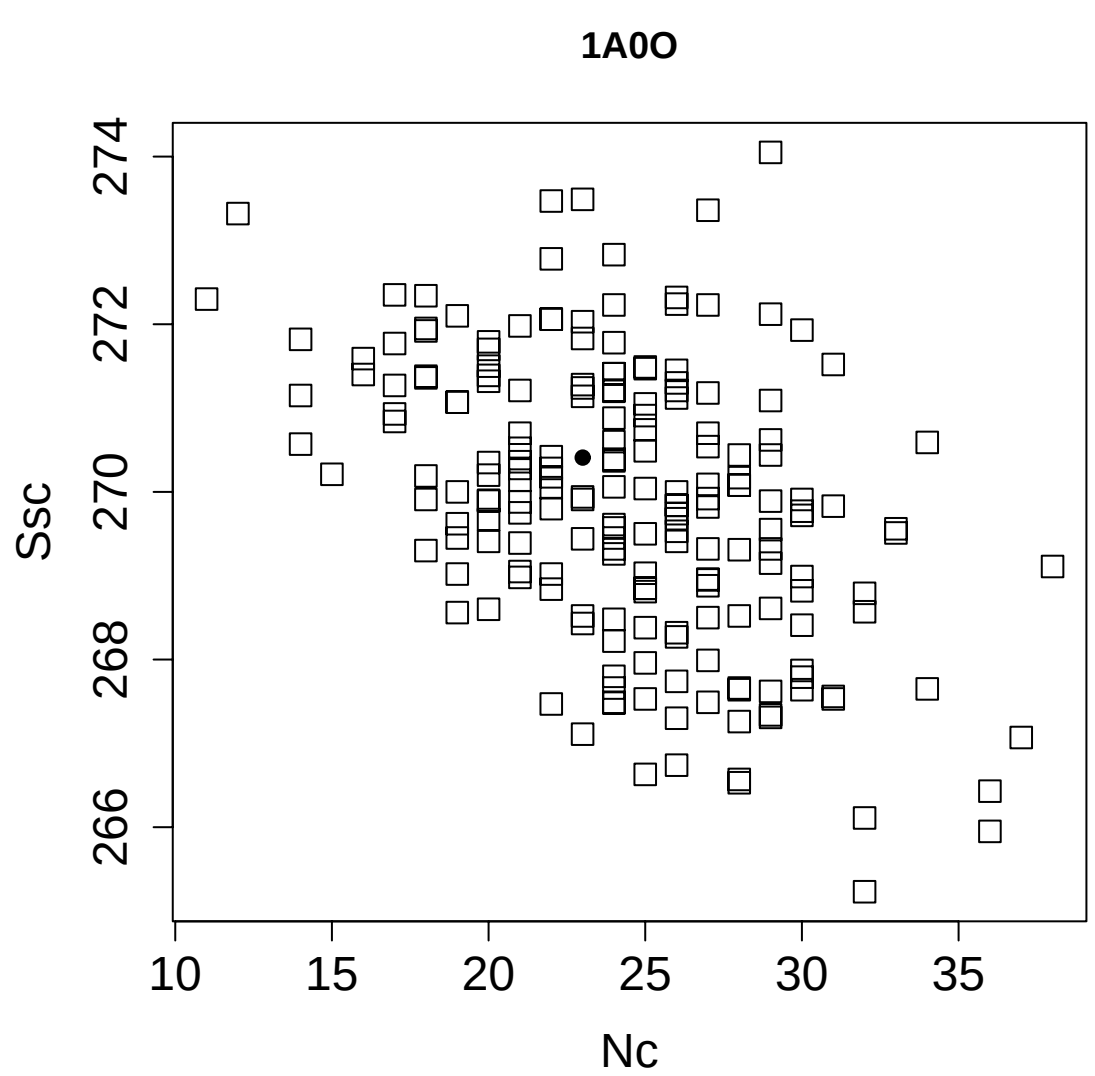
1A0O
Ssc
Nc
274
272
270
268
266
10
15
20
25
30
35

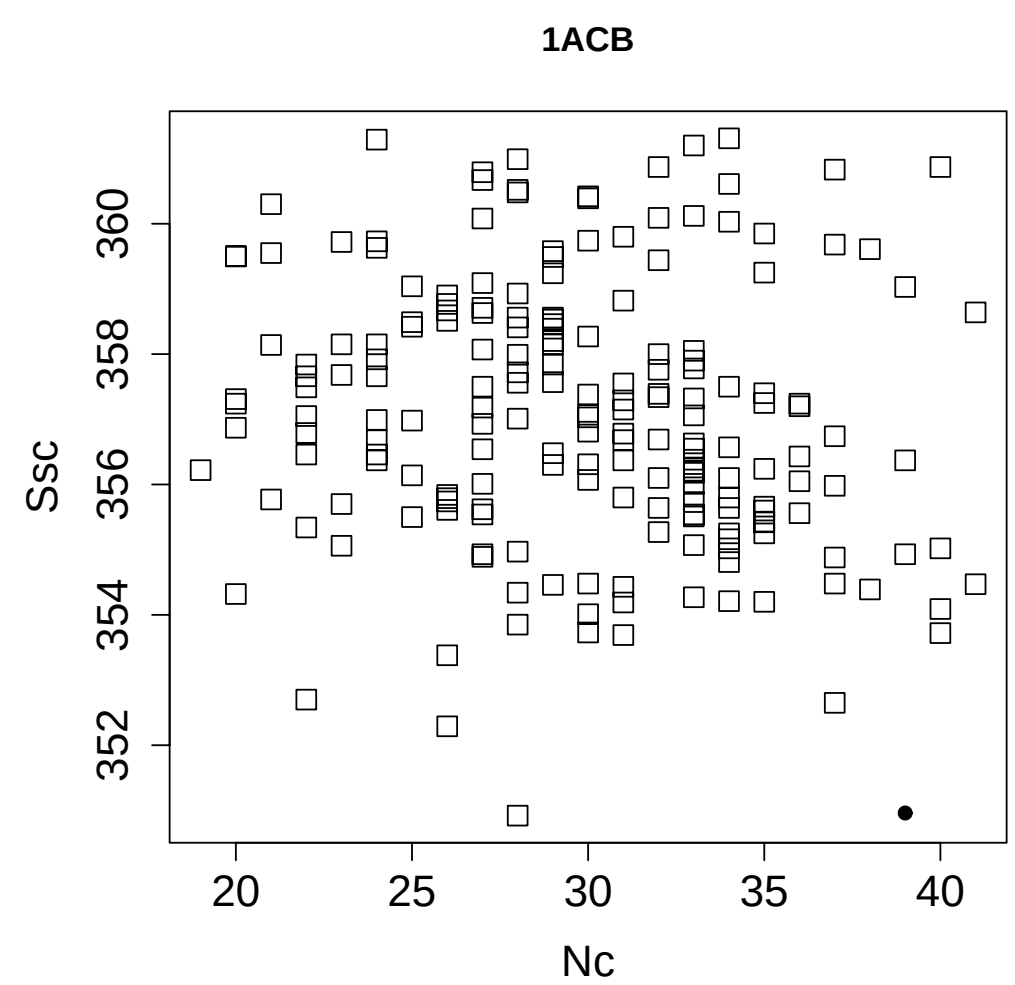
1ACB
Ssc
Nc
360
358
356
354
352
20
25
30
35
40

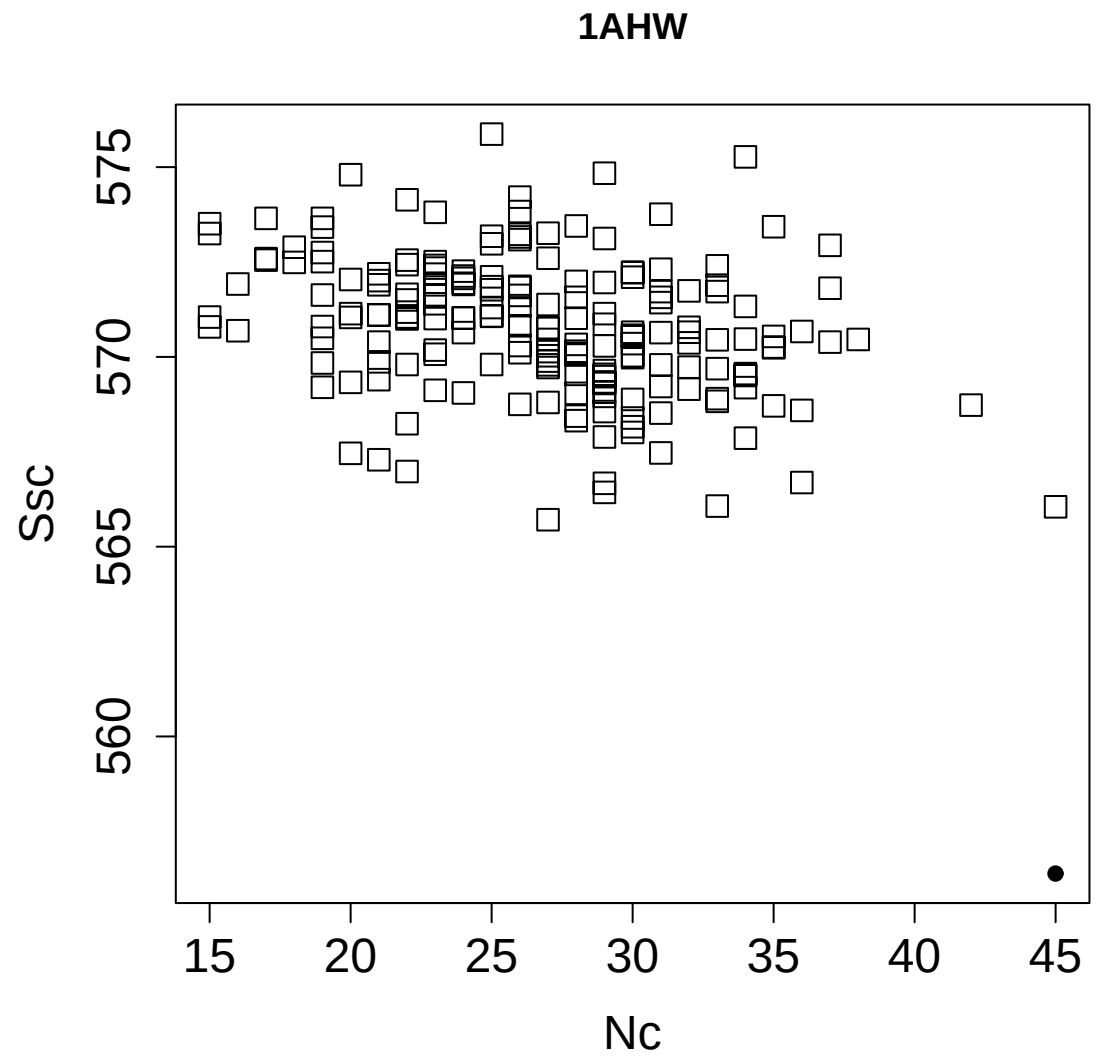
1AHW
Ssc
560
565
570
575
Nc
15
20
25
30
35
40
45

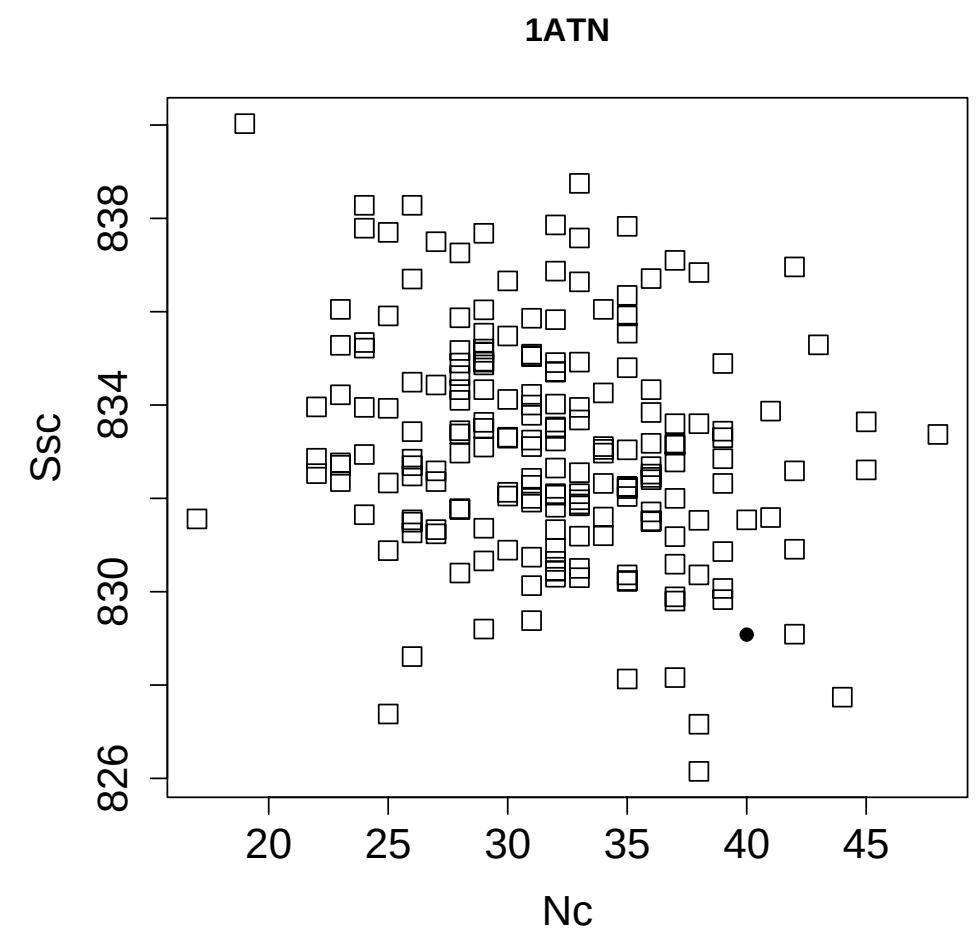
1ATN
Ssc
826
830
834
838
Nc
20
25
30
35
40
45

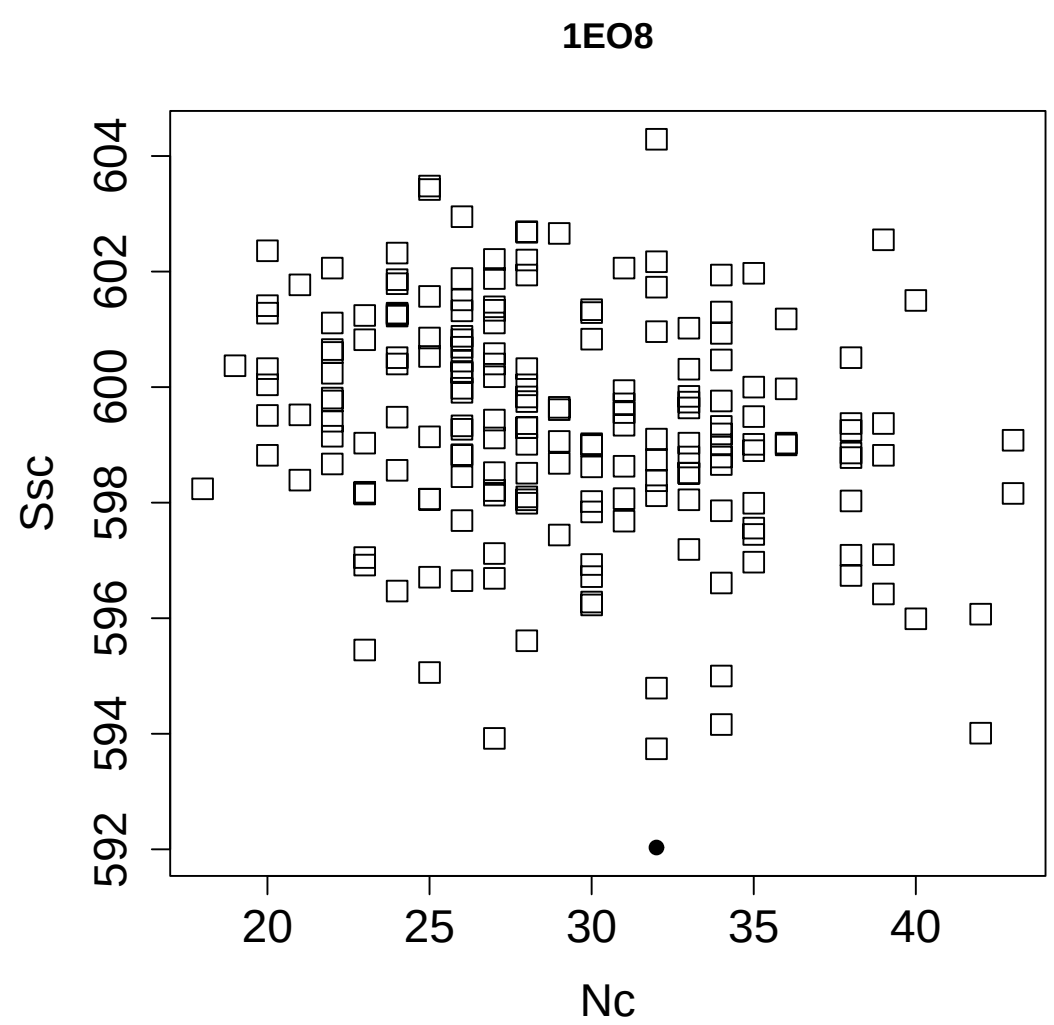
1EO8
Ssc
592
594
596
598
600
602
604
Nc
20
25
30
35
40

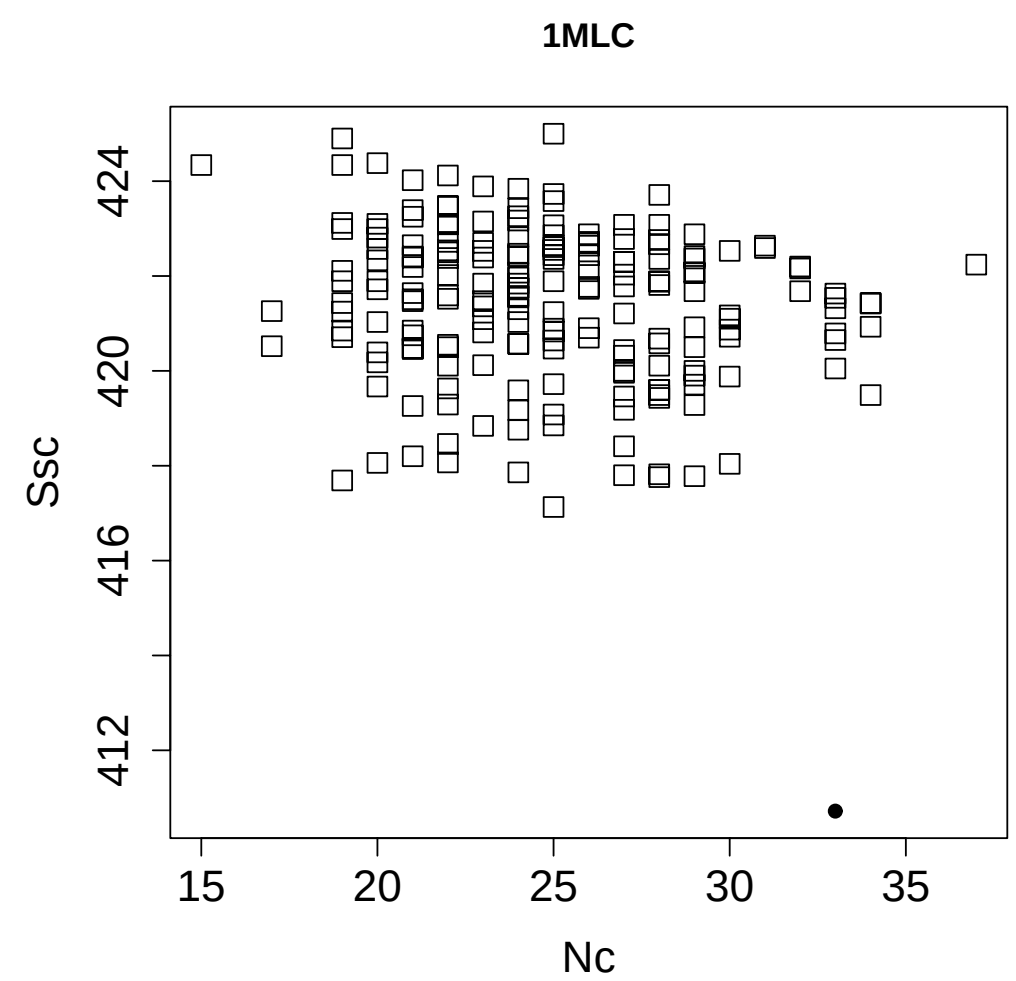
1MLC
Ssc
412
416
420
424
Nc
15
20
25
30
35

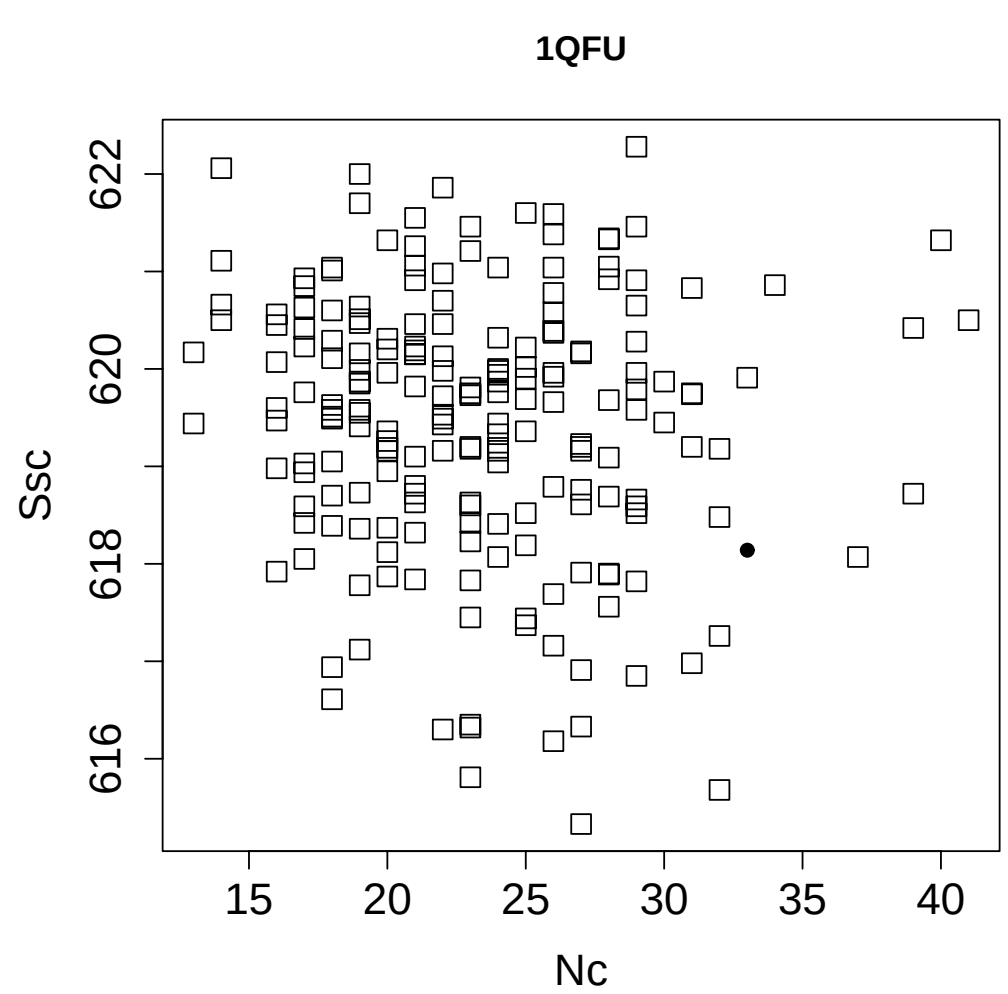
1QFU
Ssc
616
618
620
622
Nc
15
20
25
30
35
40

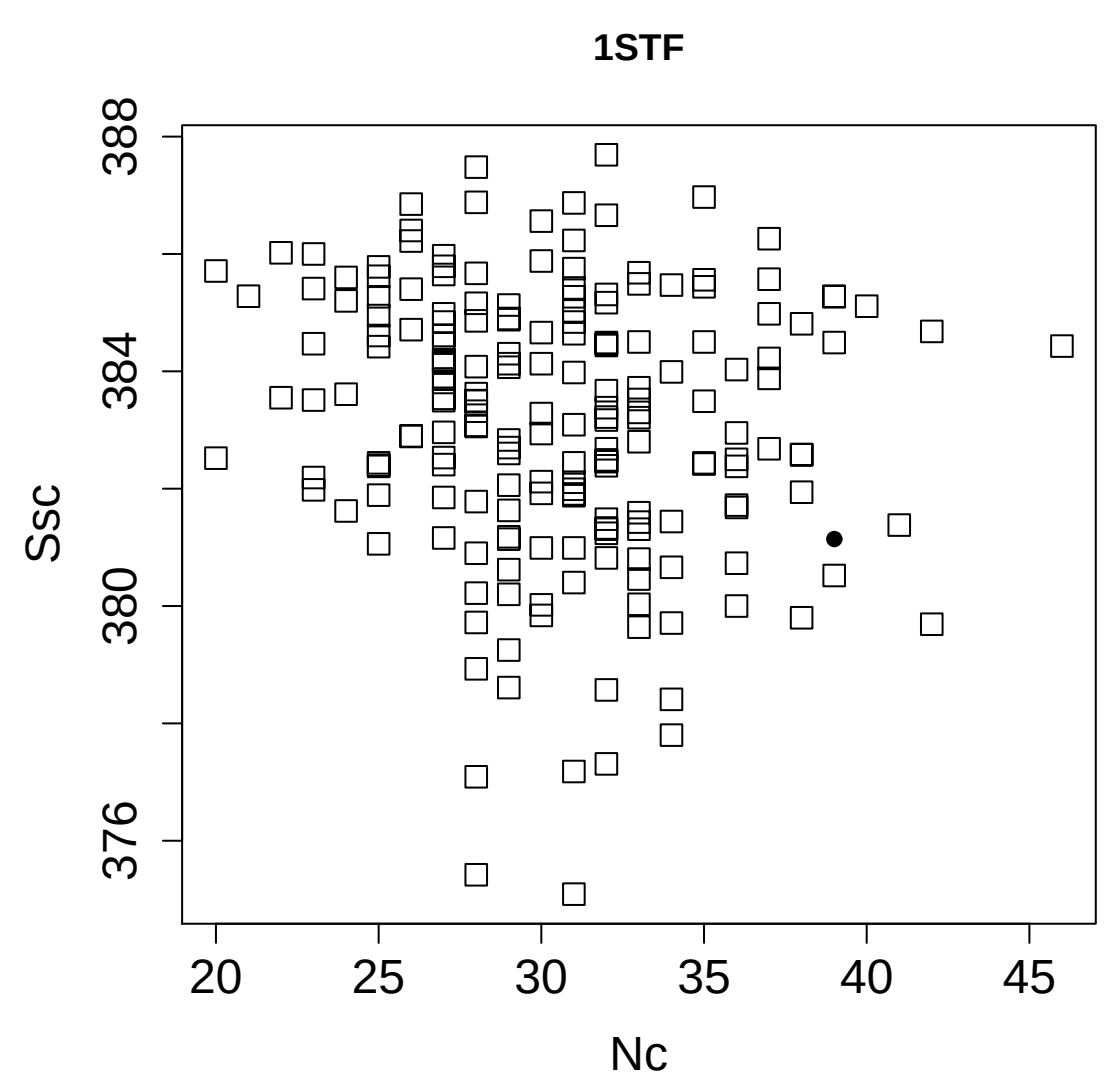
1STF
Ssc
376
380
384
388
Nc
20
25
30
35
40
45

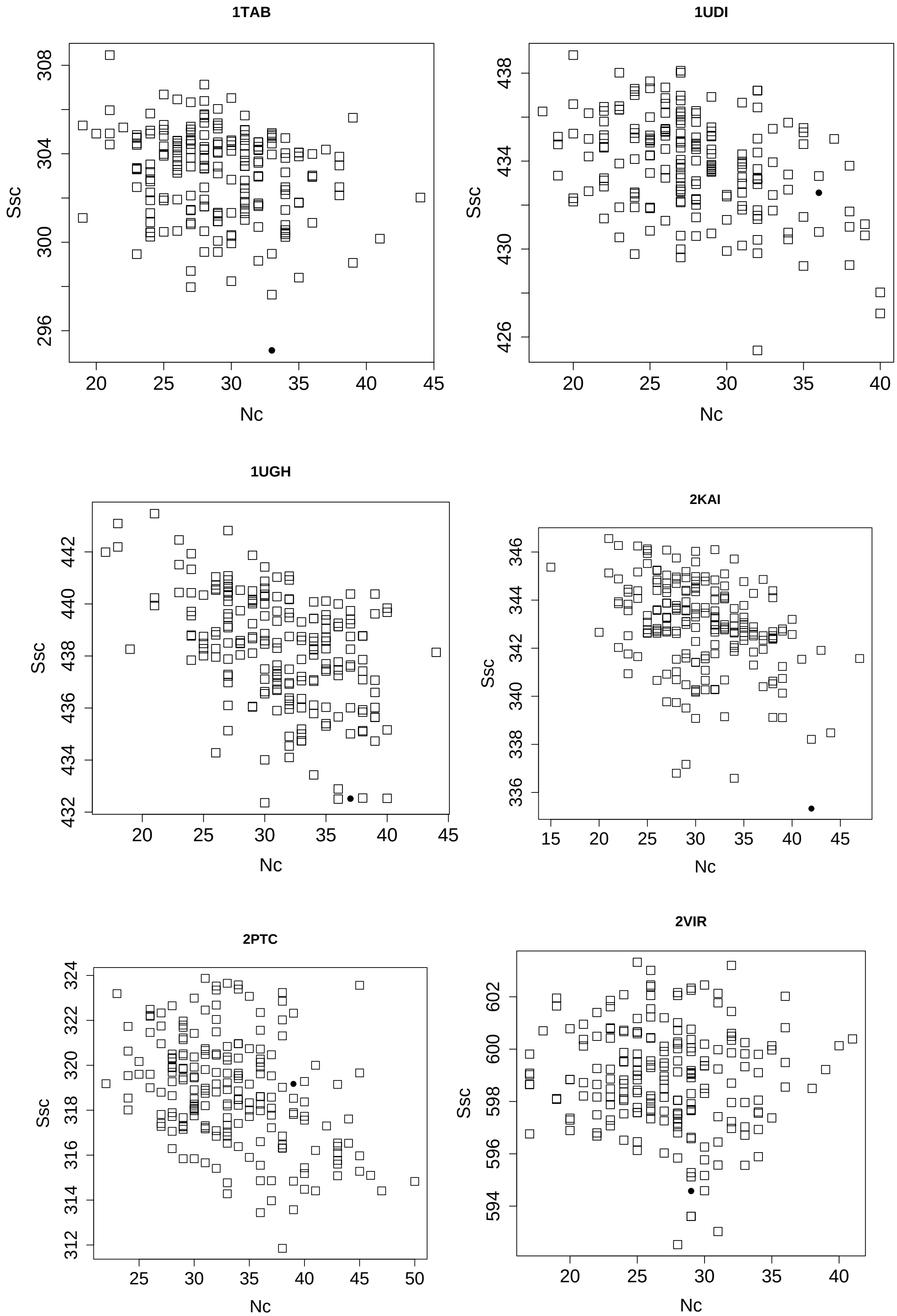
1TAB
Ssc
308
304
300
296
20
25
30
35
40
45
Nc
1UDI
Ssc
438
434
430
426
20
25
30
35
40
Nc
1UGH
Ssc
442
440
438
436
434
432
20
25
30
35
40
45
Nc
2KAI
Ssc
346
344
342
340
338
336
15
20
25
30
35
40
45
Nc
2PTC
Ssc
324
322
320
318
316
314
312
25
30
35
40
45
50
Nc
2VIR
Ssc
602
600
598
596
594
20
25
30
35
40
Nc
